# Supplementary material for: Rapid Screening and Identification of Antitumor Ingredients from the Mangrove Endophytic Fungus Using an Enzyme-Immobilized Magnetic Nanoparticulate System
Source: Molecules. 2021 Apr 13;26(8):2255. doi: 10.3390/molecules26082255 (PMC8069786; doi:10.3390/molecules26082255)
Supplement: Supplementary file 1 [file molecules-26-02255-s001.zip › Supplementary Files/supplementary.docx]

*Supplementary Material*

Rapid screening and identification of antitumor ingredients from the mangrove endophytic fungus using an enzyme-immobilized magnetic nanoparticles coupled with HPLC and MS

Nan Wei ^1^, Jun Zhao ^1^, Guimei Wu ^1^, Wenjuan Cao ^1^, Pei Luo ^3^, Zhifeng Zhang ^3^, Gang Chen ^2,4,5,^* and Lu Wen ^1,^*

^1^ School of Pharmacy, Guangdong Pharmaceutical University, Guangzhou 510006, China; [928239830@qq.com](mailto:928239830@qq.com) (N.W.); [961908856@qq.com](mailto:961908856@qq.com) (J.Z.); [2587425174@qq.com](mailto:2587425174@qq.com) (G.W.); [adaaa890@126.com](mailto:adaaa890@126.com) (W.C.)

^2^ New Drug Research and Development Center of Guangdong Pharmaceutical University, Guangzhou 510006, China

^3^ State Key Laboratory for Quality Research in Chinese Medicine, Macau University of Science and Technology, Macau 000853, China; [pluo@must.edu.mo](mailto:pluo@must.edu.mo) (P.L.); [zfzhang@must.edu.mo](mailto:zfzhang@must.edu.mo) (Z.Z.)

^4^ Guangdong Provincial Key Laboratory of Advanced Drug Delivery, Guangdong Pharmaceutical University, Guangzhou, 510006, China

^5^ Guangdong Provincial Engineering Center of Topical Precise Drug Delivery System, Guangdong Pharmaceutical University, Guangzhou, 510006, China

* Correspondence: cg753@126.com (G.C.); gywenl@163.com (L.W.)

**Table 1.** Names, MS, ^1^H and ^13^C NMR spectroscopy data of identified compounds from the crude extract of endophytic fungus *Pseudopithomyces* sp. 1512101.

| **Peak** | **Name** | **Formula** | ***m/z*** | **^1^H NMR *δ* (n H, mult, *J* in Hz)** | **^13^C NMR *δ* (ppm)** |
| --- | --- | --- | --- | --- | --- |
| **1** | cyclo-(4-hydroxyl-Pro-Leu) | C_11_H_18_N_2_O_3_ | 227.2 [M+H]^+^ | 4.53 (2H, m, H-4,6), 4.18 (1H, d, *J* = 4.0 Hz, H-9), 3.68 (1H, dd, *J* = 4.0, 8.0 Hz, H-3), 3.45 (1H, d, *J* = 12.0 Hz, H-3), 2.30 (1H, dd, *J* = 4.0, 8.0 Hz, H-5), 2.09 (1H, m, H-5), 1.89 (2H, m, H-10), 1.53 (1H, dd, *J* = 4.0, 8.0 Hz, H-10), 1.33 (1H, d, *J* = 12.0 Hz, H-11), 0.98 (6H, d, *J* = 4.0 Hz, H-12,13). | 173.1 (C-7), 169.0 (C-1), 69.1 (C-4), 58.7 (C-6), 55.2 (C-3), 54.6 (C-9), 39.4 (C-10), 38.2 (C-5), 25.8 (C-11), 23.3 (C-12), 22.2 (C-13). |
| **2** | cyclo-(Pro-Val) | C_10_H_16_N_2_O_2_ | 197.2 [M+H]^+^ | 4.59 (1H, s, H-8), 4.23 (1H, t, *J* = 8.0, 8.0 Hz, H-6), 4.06 (1H, s, H-9), 3.57 (2H, m, H-3), 2.52 (1H, m, H-10), 2.35 (1H, m, H-5), 2.00 (3H, m, H-4,5), 1.12 (3H, d, *J* = 8.0 Hz, H-12), 0.95 (3H, d, *J* = 4.0 Hz, H-11). | 172.6 (C-7), 167.6 (C-1), 61.5 (C-9), 60.0 (C-6), 46.2 (C-3), 29.9 (C-10), 29.5 (C-5), 23.3 (C-4), 18.8 (C-12), 16.7 (C-11). |
| **3** | Fusaristatin C | C_25_H_43_N_3_O_6_ | 482.32259 [M+H]^+^ | 5.80 (1H, s, H-23), 5.45 (1H, s, H-23), 5.08 (1H, m, H-3), 4.62 (1H, t, *J* = 5.0, 5.0 Hz, H-16), 3.82 (1H, m, H-20), 3.80 (1H, m, H-17), 3.76 (1H, dd, *J* = 5.0, 10.0 Hz, H-17), 3.42 (1H, m, H-20), 2.77 (1H, dt, *J* = 5.0, 10.0, 15.0 Hz, H-2), 2.47 (1H, m, H-19), 2.32 (1H, dt, *J* = 5.0, 5.0, 15.0 Hz, H-19), 1.78 (1H, m, H-4), 1.69 (1H, m, H-4), 1.42 (1H, m, H-11), 1.32 (14H, s, H-5~10,12,13), 1.20 (3H, d, *J* = 5.0 Hz, H-5,10), 1.20 (2H, m, 2-CH_3_), 0.92 (3H, t, *J* = 5.0, 10.0 Hz, H-14), 0.91 (3H, d, *J* = 5.0 Hz, 11-CH_3_). | 175.4 (C-1), 174.0 (C-18), 171.1 (C-15), 166.5 (C-21), 138.3 (C-22), 114.6 (C-23), 77.7 (C-3), 62.3 (C-17), 55.5 (C-16), 44.9 (C-2), 38.2 (C-10), 37.3 (C-19), 37.0 (C-20), 33.8 (C-11), 33.1 (C-12), 32.6 (C-5), 31.1 (C-6), 30.8 (C-7), 30.5 (C-4), 30.5 (C-8), 28.1 (C-9), 23.7 (C-13), 19.8 (11-CH_3_), 15.3 (2-CH_3_), 14.4 (C-14). |
| **4** | Ergosterol Peroxide | C_28_H_44_O_3_ | - | 6.50 (1H, d, *J* = 8.0 Hz, H-7), 6.24 (1H, d, *J* = 8.0 Hz, H-6), 5.22 (1H, dd, *J* = 8.0, 16.0 Hz, H-23), 5.14 (1H, dd, *J* = 8.0, 16.0 Hz, H-22), 3.97 (1H, m, H-3), 1.00 (3H, d, *J* = 4.0 Hz, H-20), 0.91 (3H, d, *J* = 4.0 Hz, H-28), 0.88 (3H, s, H-19), 0.84~0.81 (9H, m, H-18,26,27). | - |
| **5** | Ergosterol | C_28_H_44_O | - | 5.57 (1H, m, H-6), 5.38 (1H, m, H-7), 5.19 (2H, m, H-22,23), 3.63 (1H, m, H-3), 1.04 (3H, d, *J* = 4.0 Hz, H-21), 0.94 (3H, s, H-19), 0.92 (3H, d, *J* = 4.0 Hz, H-28), 0.83 (6H, m, H-26,27), 0.63 (3H, s, H-18). | 141.5 (C-8), 139.9 (C-5), 135.7 (C-22), 132.1 (C-23), 119.7 (C-6), 116.4 (C-7), 70.6 (C-3), 55.9 (C-17), 54.7 (C-14), 46.4 (C-9), 43.0 (C-13), 41.0 (C-4), 40.6 (C-24), 39.3 (C-12), 38.5 (C-1), 37.2 (C-10), 33.2 (C-25), 32.2 (C-2), 28.4 (C-16), 23.2 (C-15), 21.3 (C-11), 20.1 (C-26), 19.8 (C-27), 17.8 (C-28), 16.4 (C-19), 12.2 (C-18). |
| **6** | Cerevisterol | C_28_H_46_O_3_ | - | 5.24 (1H, dd, *J* = 8.0, 16.0 Hz, H-23), 5.17 (1H, dd, *J* = 8.0, 16.0 Hz, H-22), 5.08 (1H, m, H-7), 4.48 (1H, d, *J* = 4.0 Hz, 6-OH), 4.21 (1H, d, *J* = 8.0 Hz, 5-OH), 3.77 (1H, m, H-3), 3.57 (1H, s, H-6), 0.99 (3H, d, *J* = 4.0 Hz, H-21), 0.91 (3H, s, H-19), 0.89 (3H, d, *J* = 4.0 Hz, H-28), 0.82 (3H, d, *J* = 4.0 Hz, H-27), 0.80 (3H, d, *J* = 4.0 Hz, H-26), 0.55 (3H, s, H-18). | 139.6 (C-8), 135.4 (C-22), 131.4 (C-23), 119.4 (C-7), 74.4 (C-5), 72.1 (C-6), 65.9 (C-3), 55.3 (C-17), 54.2 (C-14), 43.0 (C-13), 42.2 (C-9), 42.0 (C-24), 40.2 (C-4,20), 38.9 (C-12), 36.6 (C-10), 32.4 (C-2,25), 31.2 (C-1), 27.7 (C-16), 22.6 (C-15), 21.3 (C-11), 20.9 (C-21), 19.7 (C-26), 19.4 (C-27), 17.7 (C-19), 17.3 (C-28), 12.0 (C-18). |


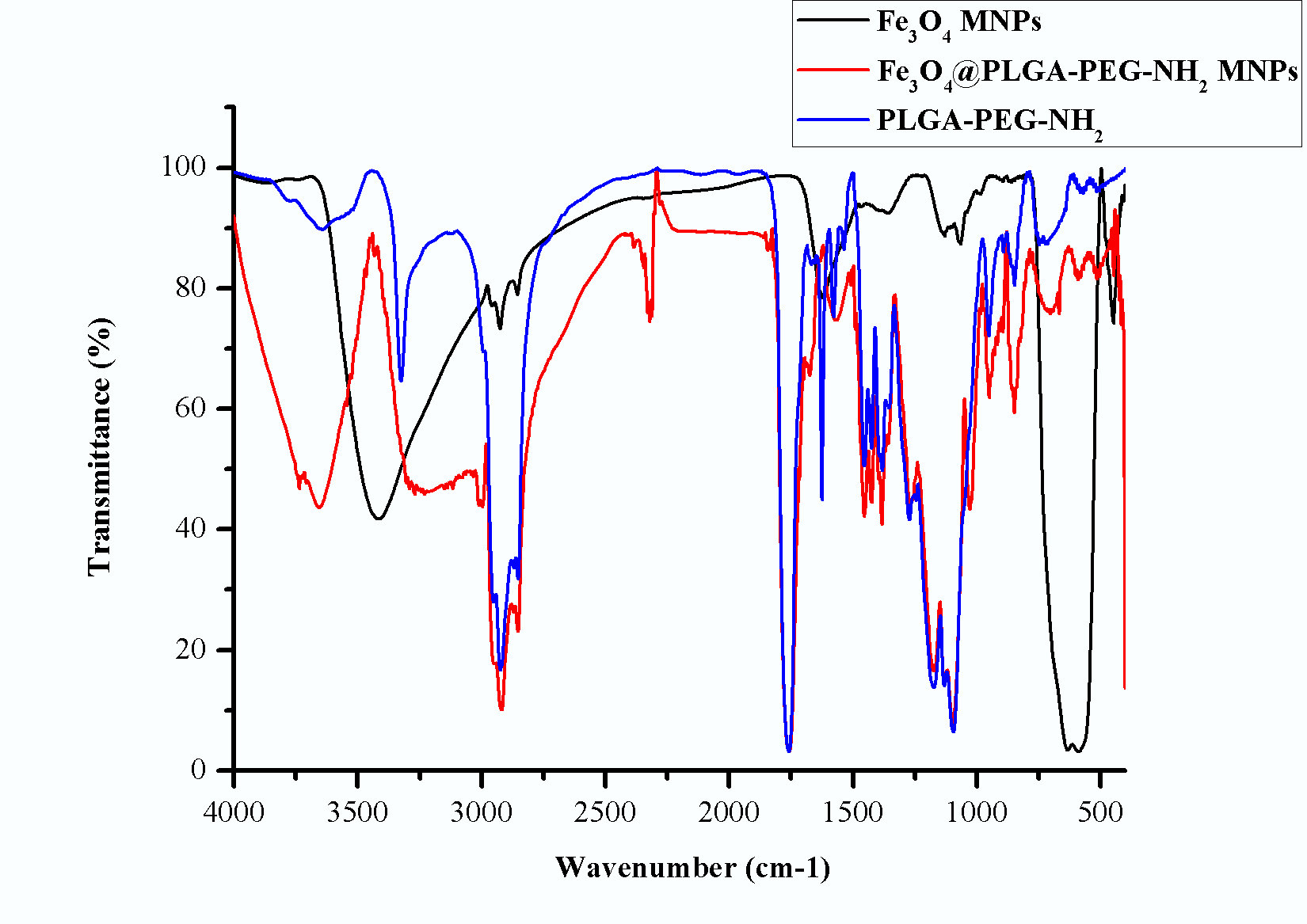


**Figure 1.** FTIR spectrum of Fe_3_O_4_@PLGA-PEG-NH_2_ MNPs.


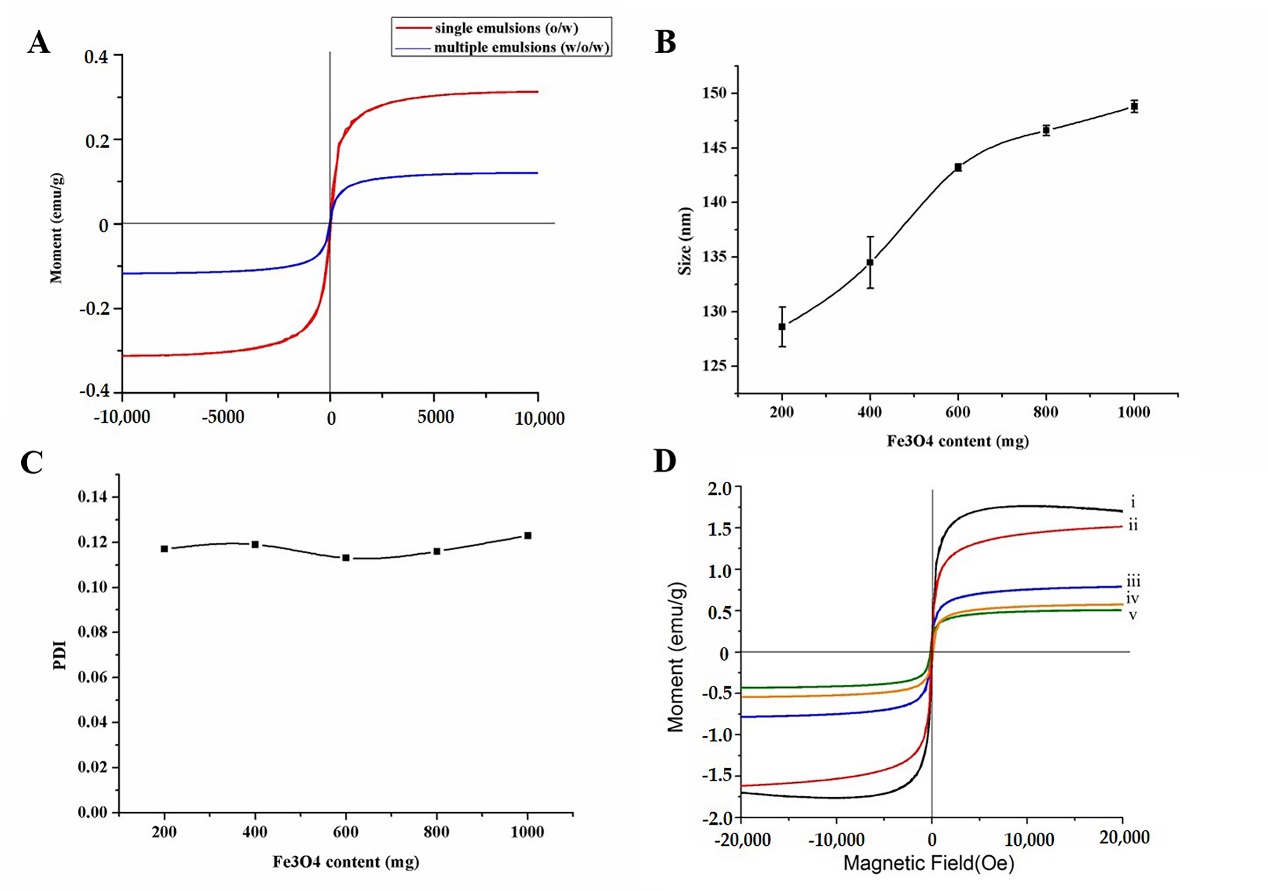


**Figure 2.** (A) Hysteresis loops of Fe_3_O_4_@PLGA-PEG-NH_2_ MNPs by single emulsions (o/w) and multiple emulsions (w/o/w); size (B), PDI (C) and hysteresis loops (D) of Fe_3_O_4_@PLGA-PEG-NH_2_ MNPs with different amounts of Fe_3_O_4_ MNPs.


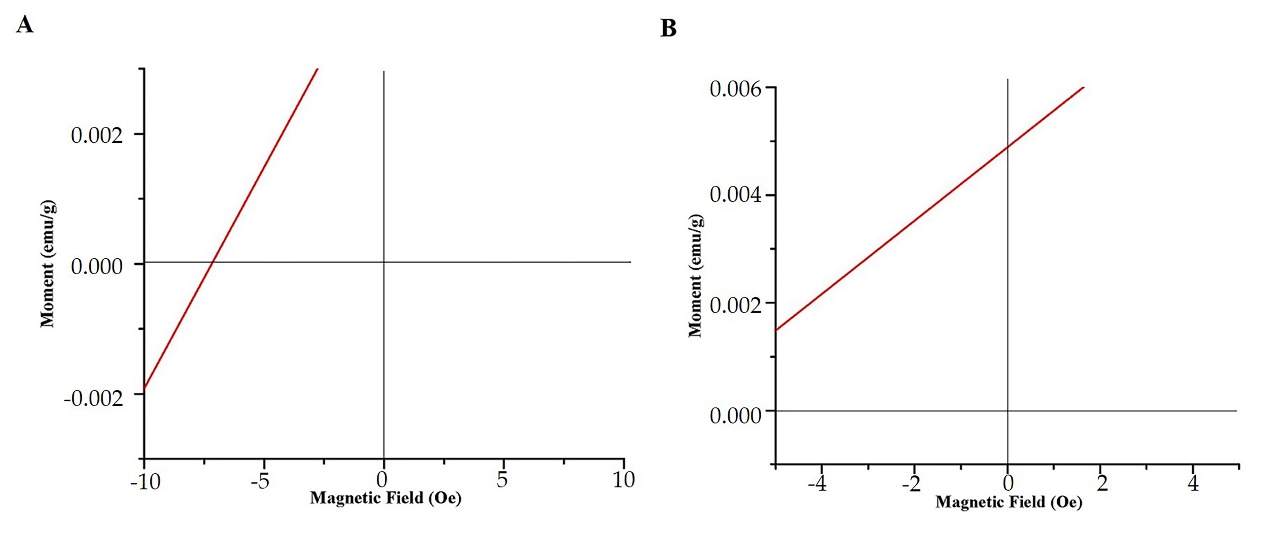


**Figure 3.** (**A**) Coercive force of Fe_3_O_4_@PLGA-PEG-NH_2_ MNPs; (**B**) Remanence of Fe_3_O_4_@PLGA-PEG-NH_2_ MNPs.

**Figure 4.** UV-vis absorption spectra of PLA_2_- MNPs and Fe_3_O_4_@PLGA-PEG-NH_2_ MNPs.


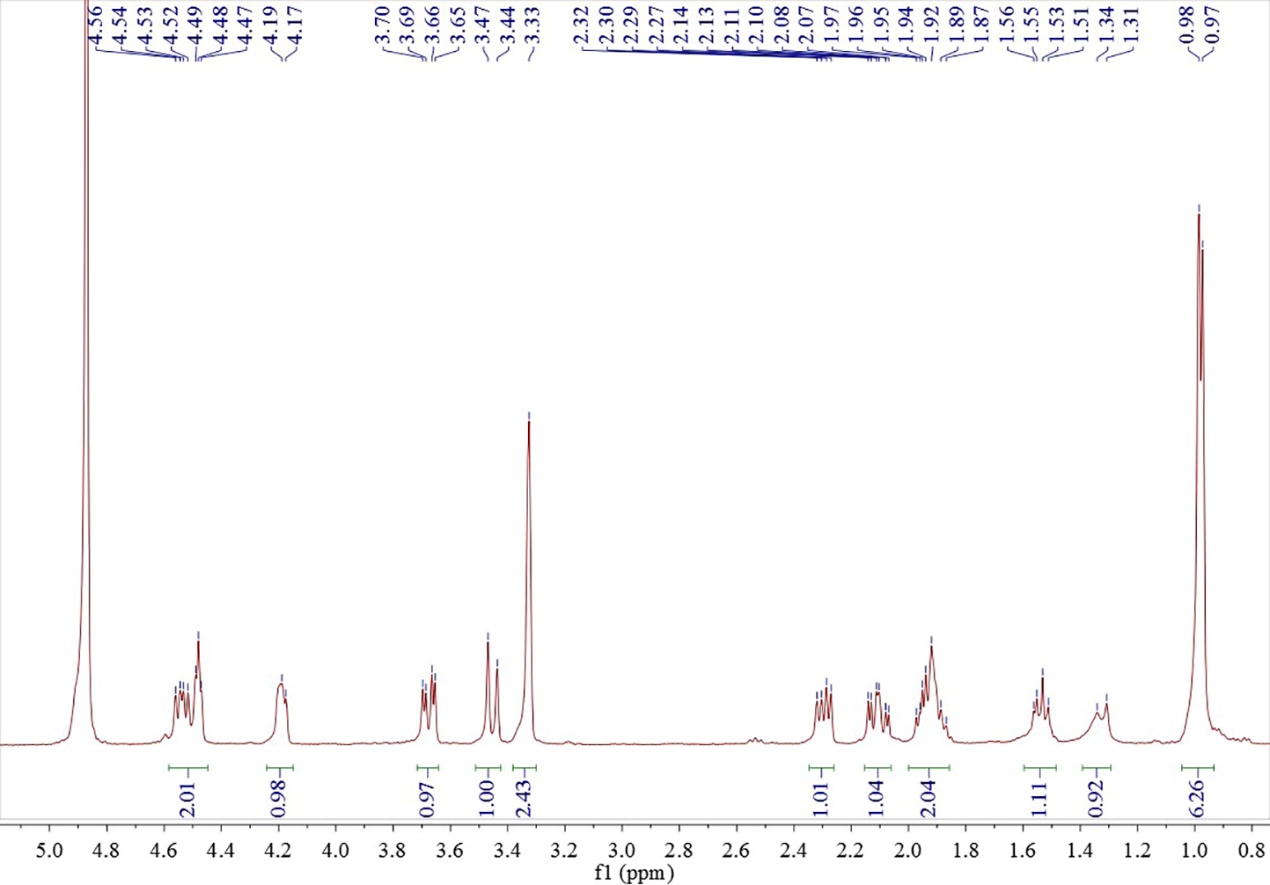


**Figure 5.** ^1^H-NMR spectra of compound **1**.


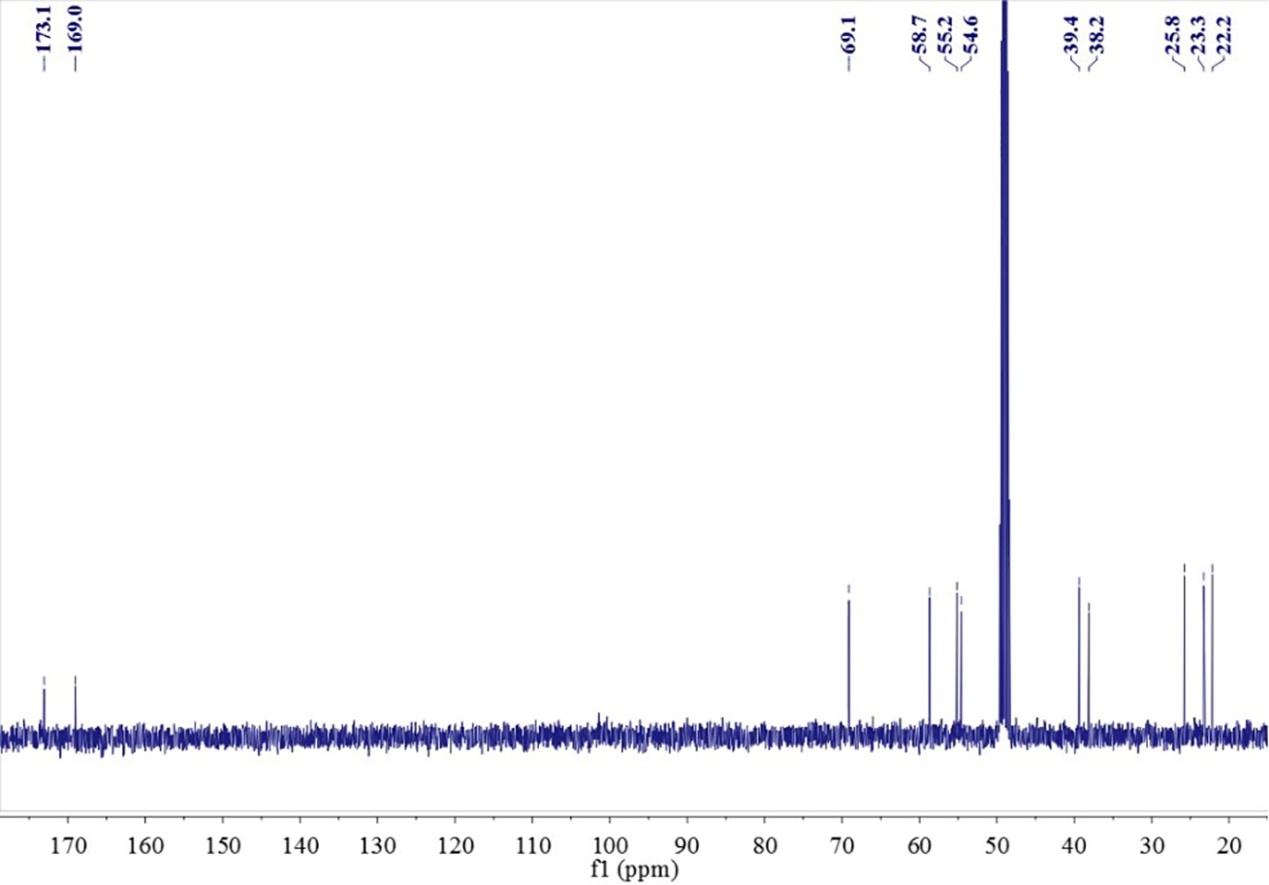


**Figure 6.** ^13^C-NMR spectra of compound **1**.


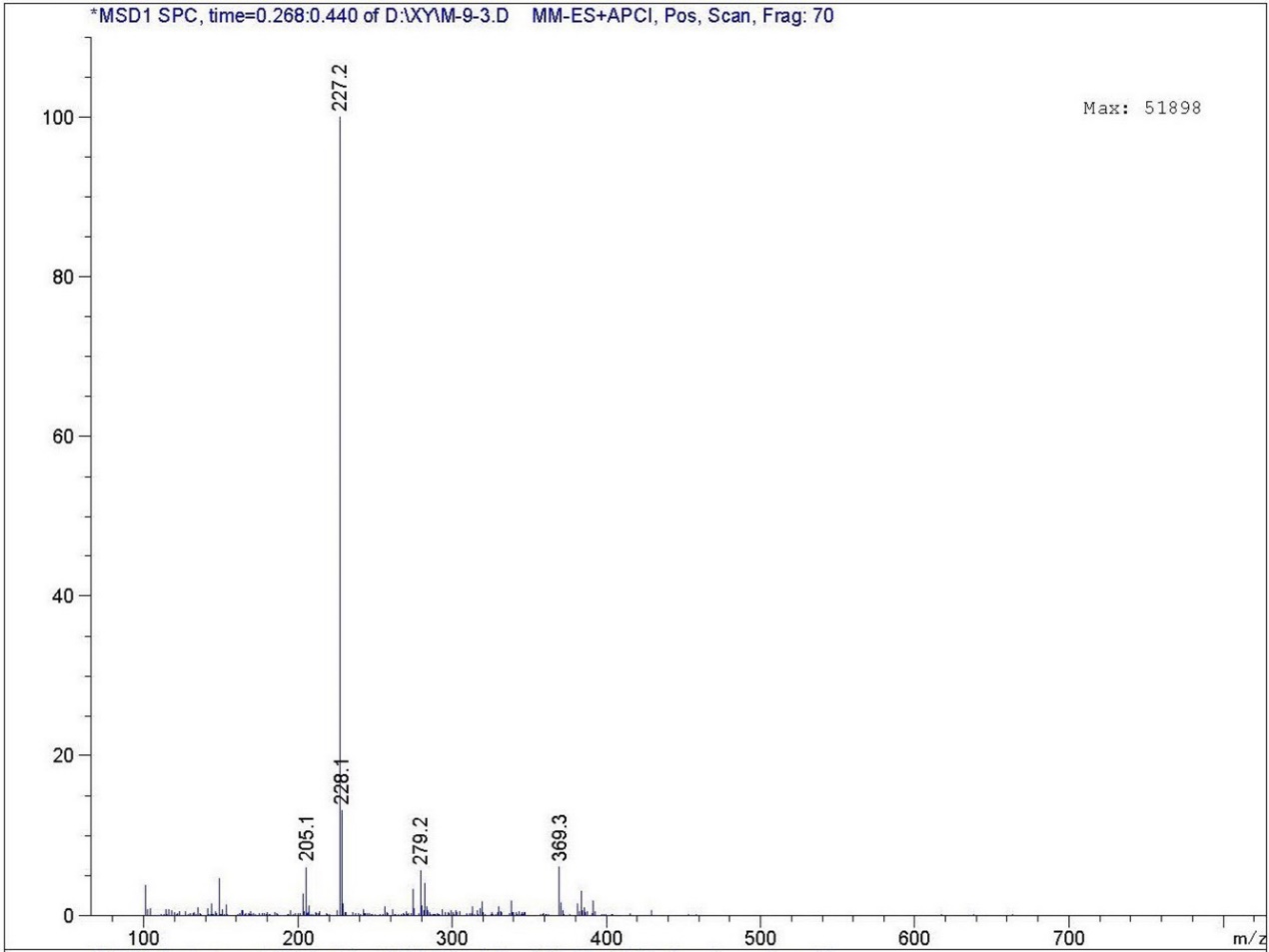


**Figure 7.** MS Spectra of compound **1**.


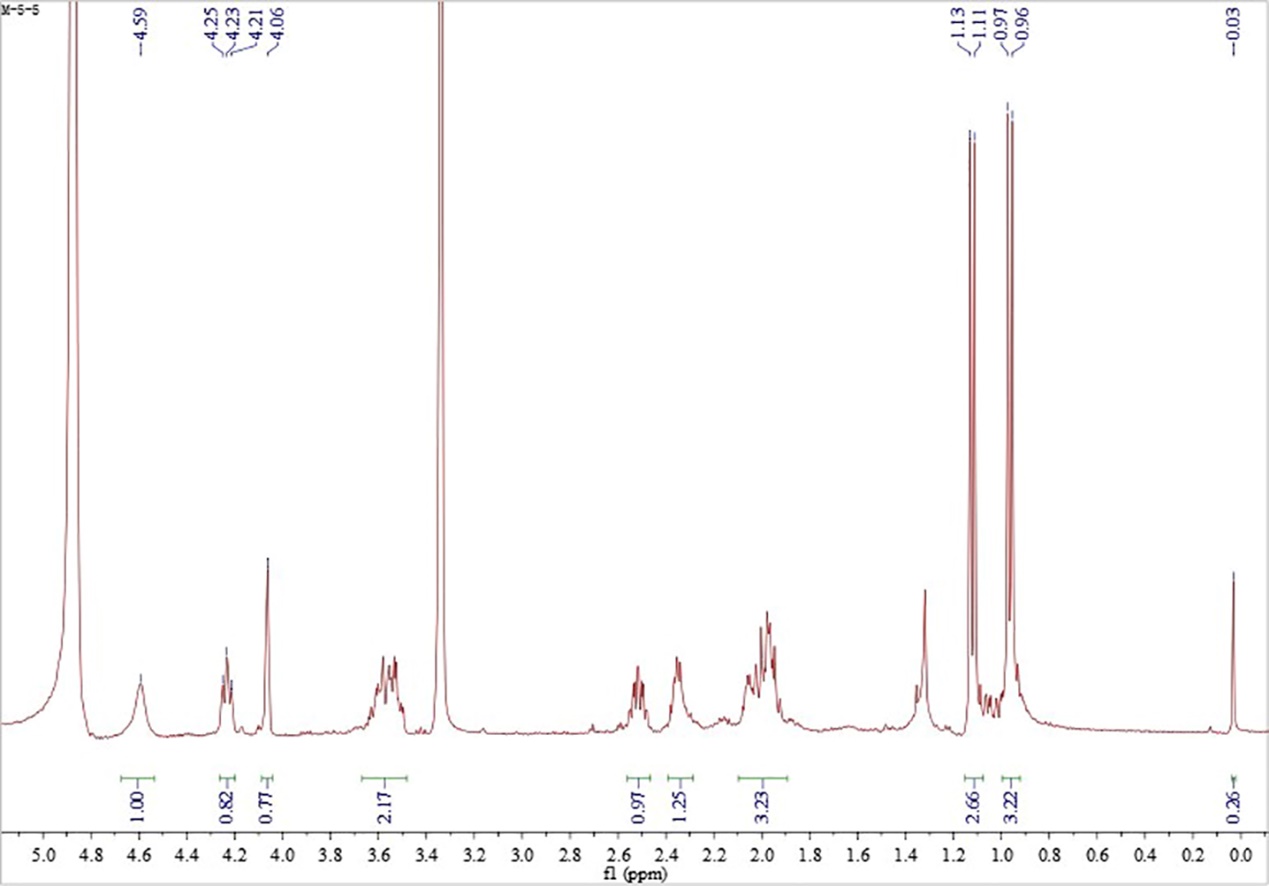


**Figure 8.** ^1^H-NMR spectra of compound **2**.


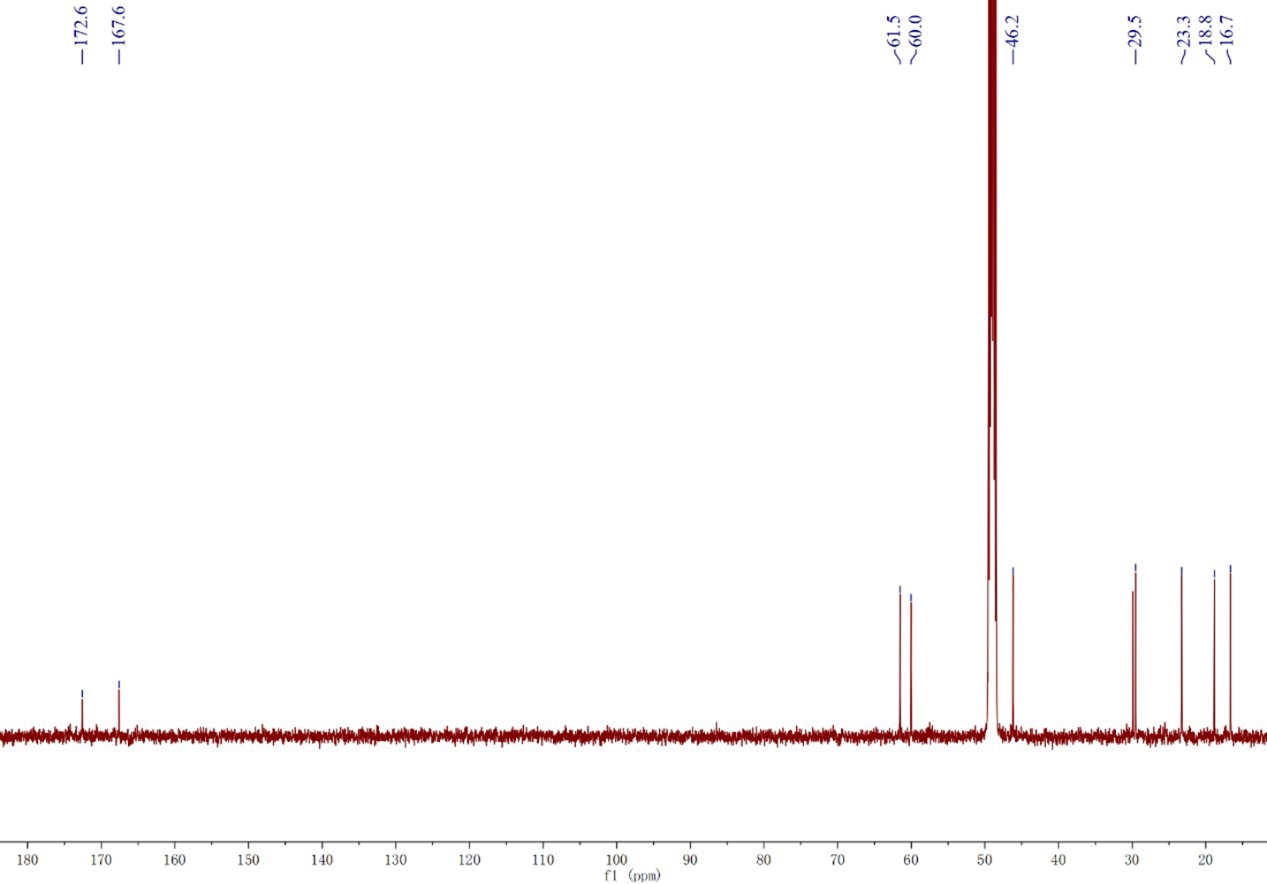


**Figure 9.** ^13^C-NMR spectra of compound **2**.


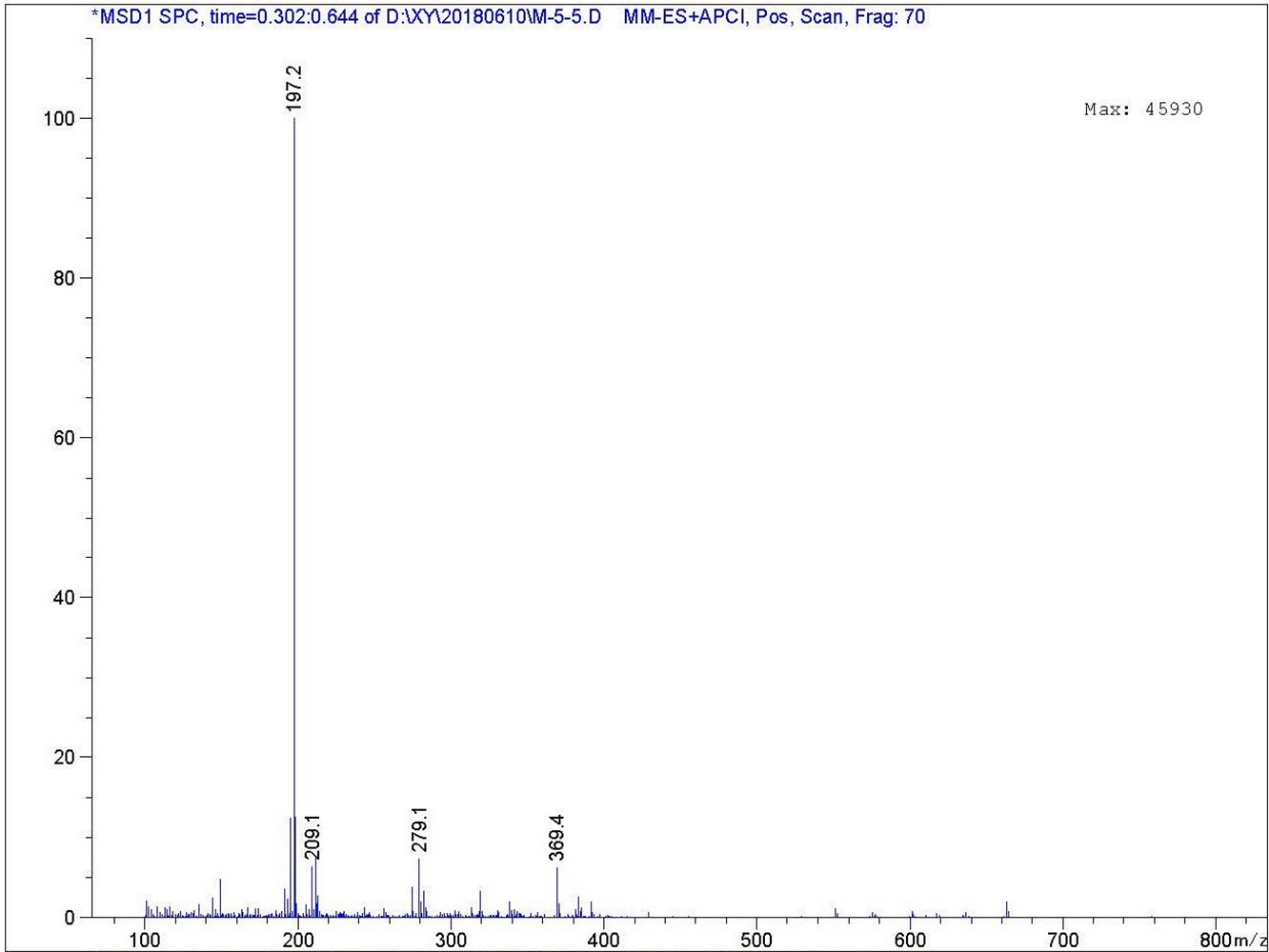


**Figure 10.** MS Spectra of compound **2**.


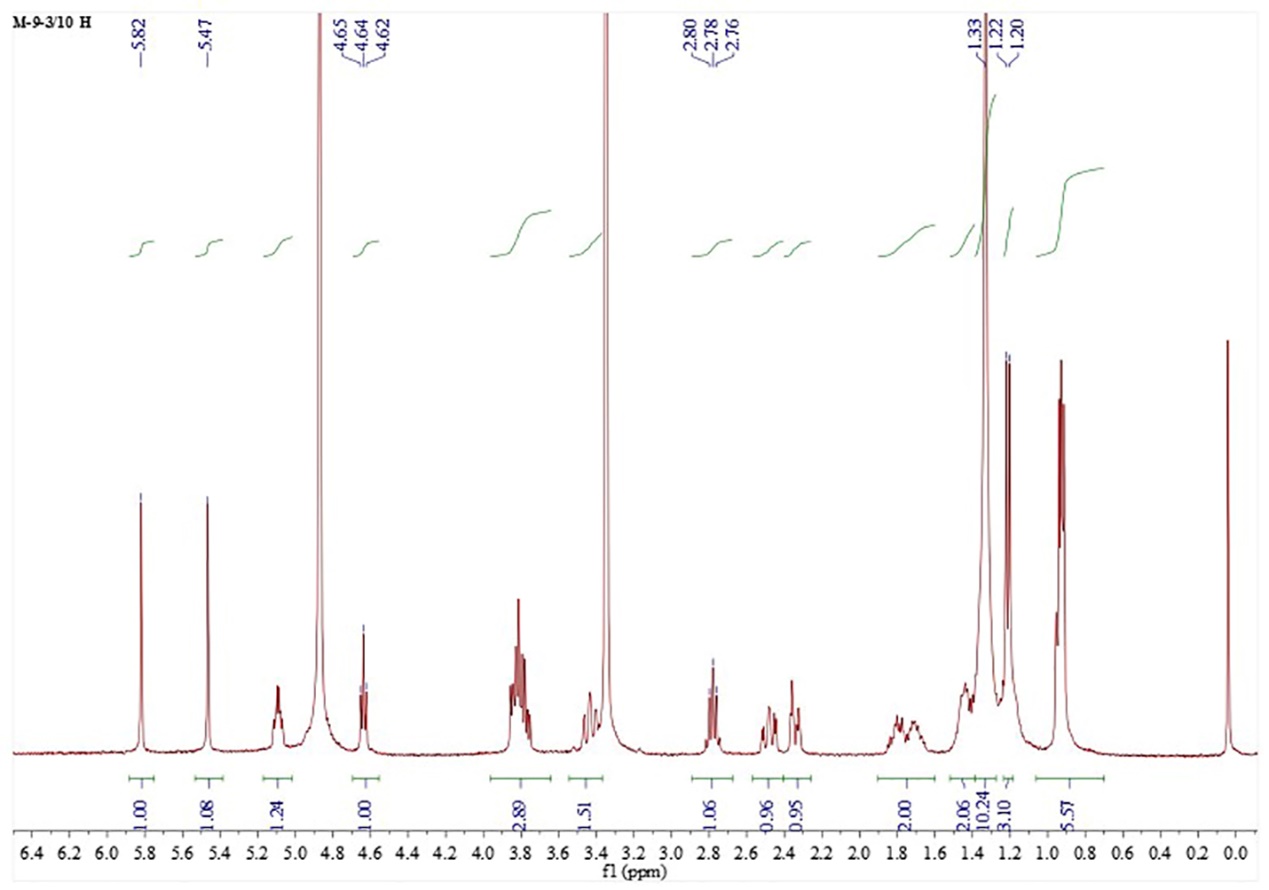


**Figure 11.** ^1^H-NMR spectra of compound **3**.


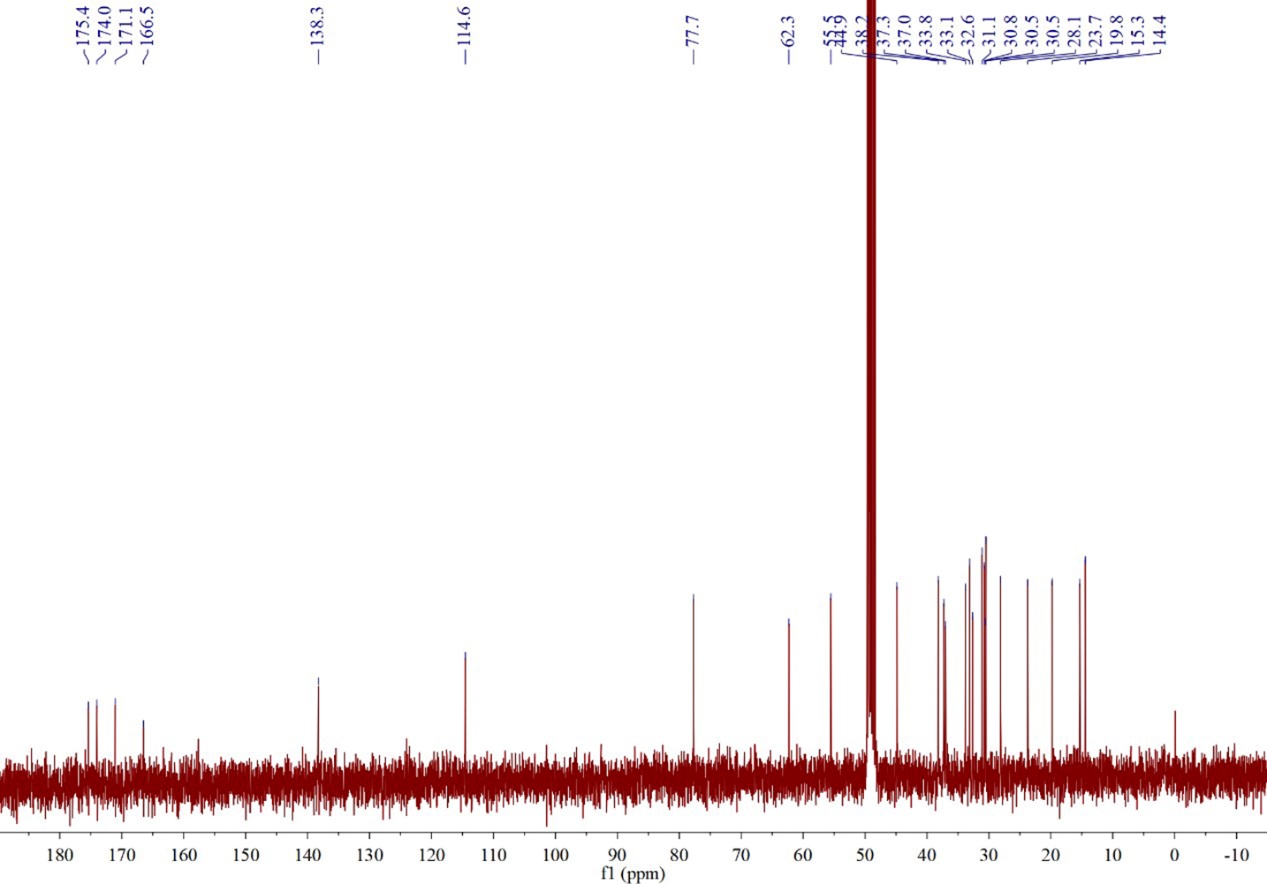


**Figure 12.** ^13^C-NMR spectra of compound **3**.


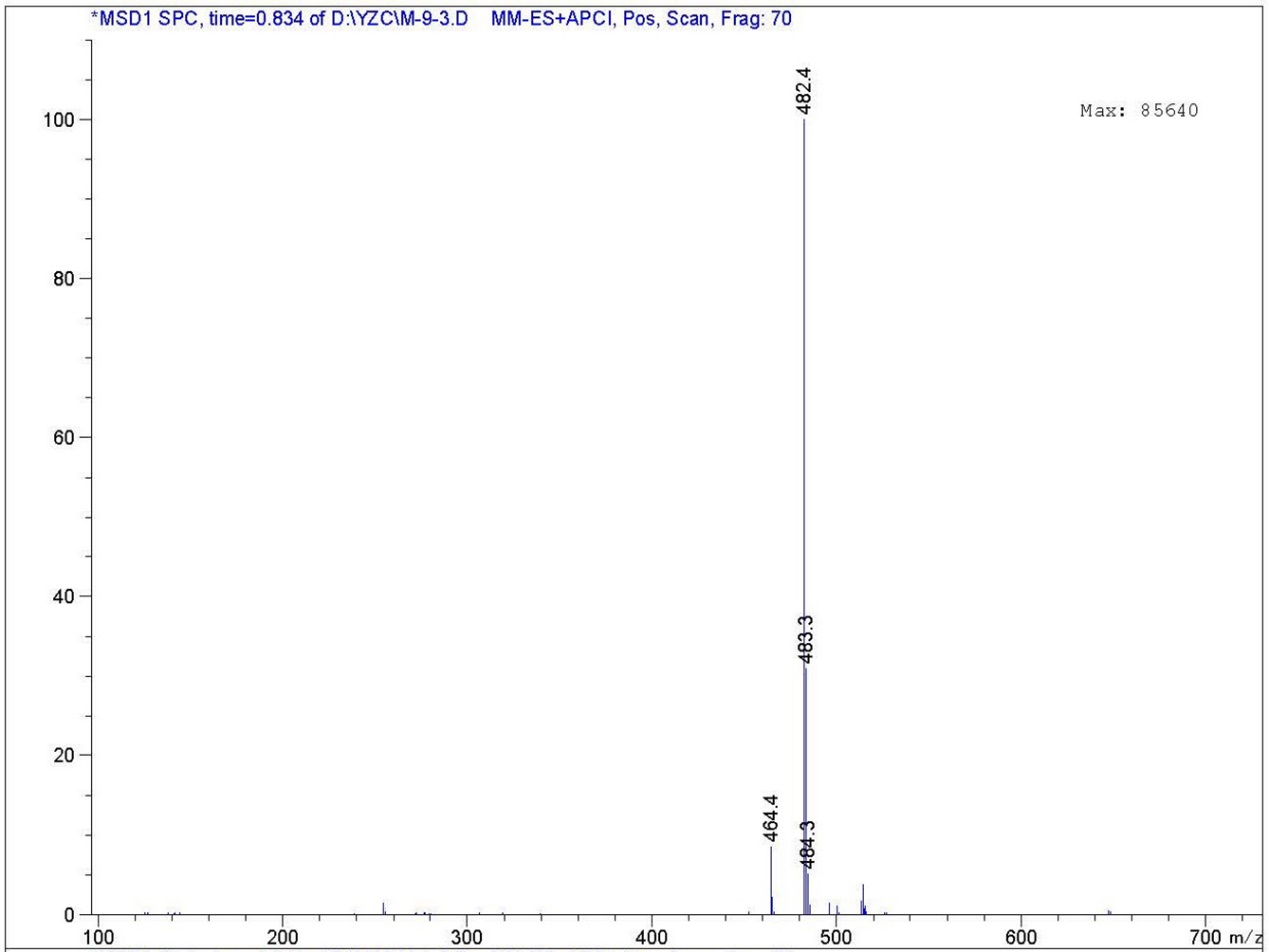


**Figure 13.** MS Spectra of compound **3**.


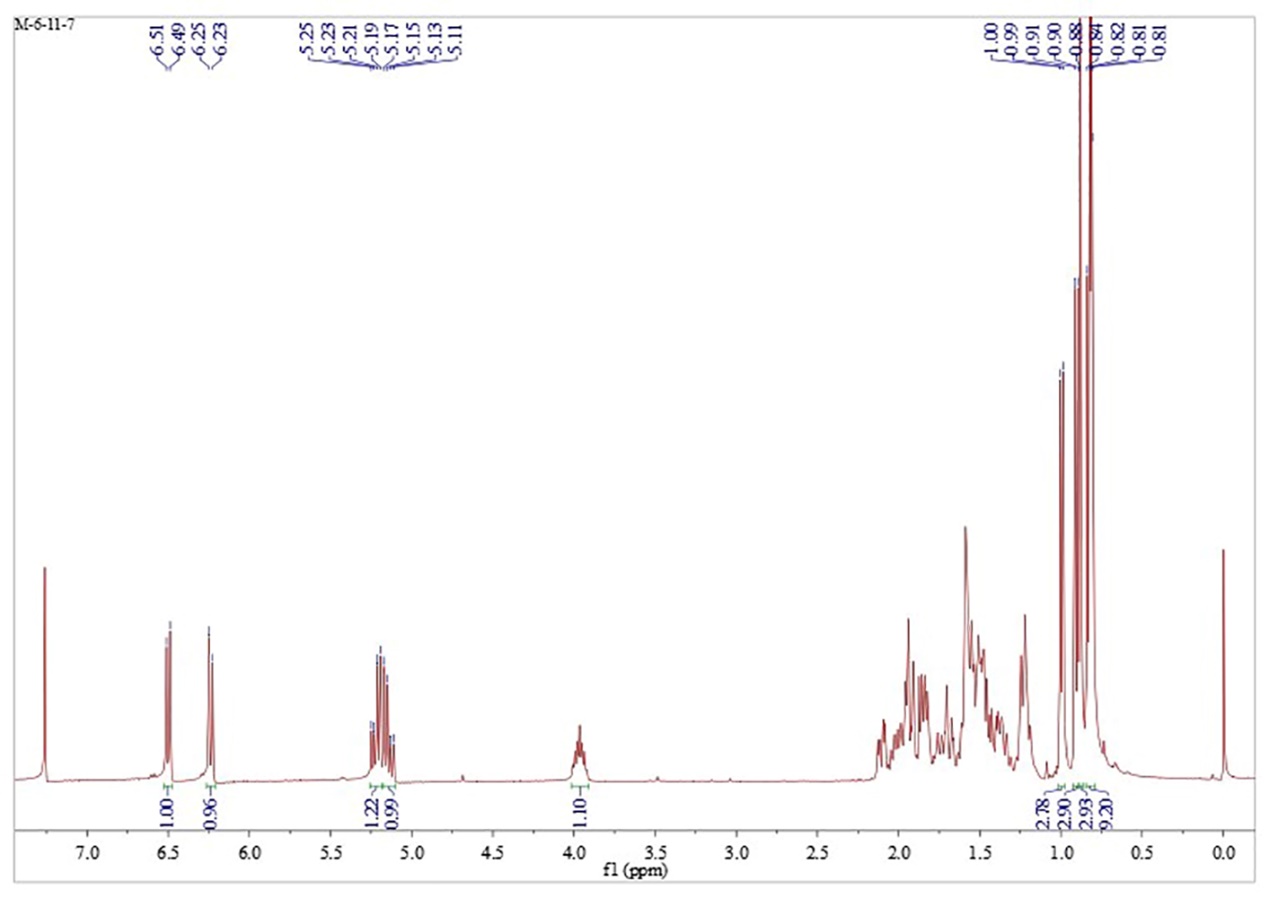


**Figure 14.** ^1^H-NMR spectra of compound **4**.


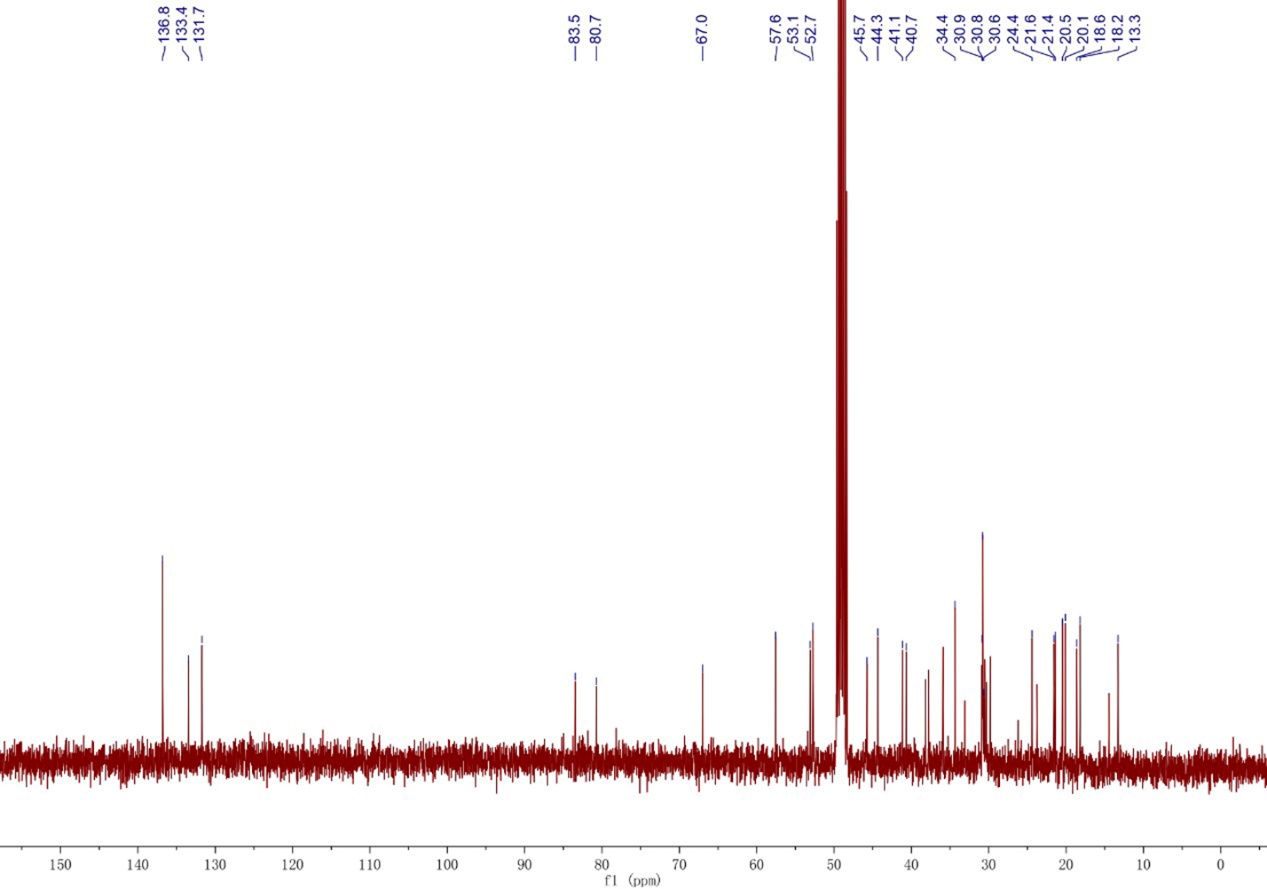


**Figure 15.** ^13^C-NMR spectra of r compound **4**.


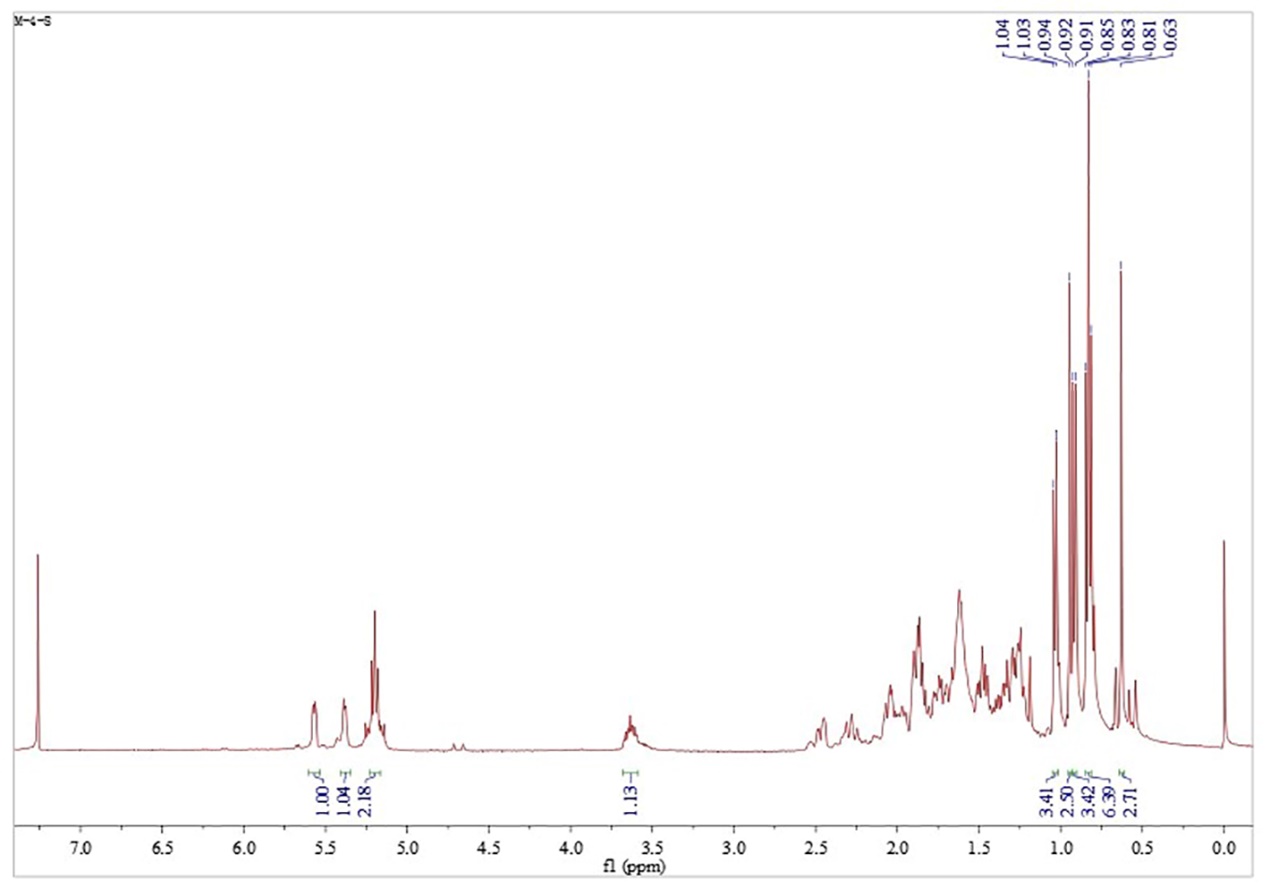


**Figure 16.** ^1^H-NMR spectra of compound **5**.


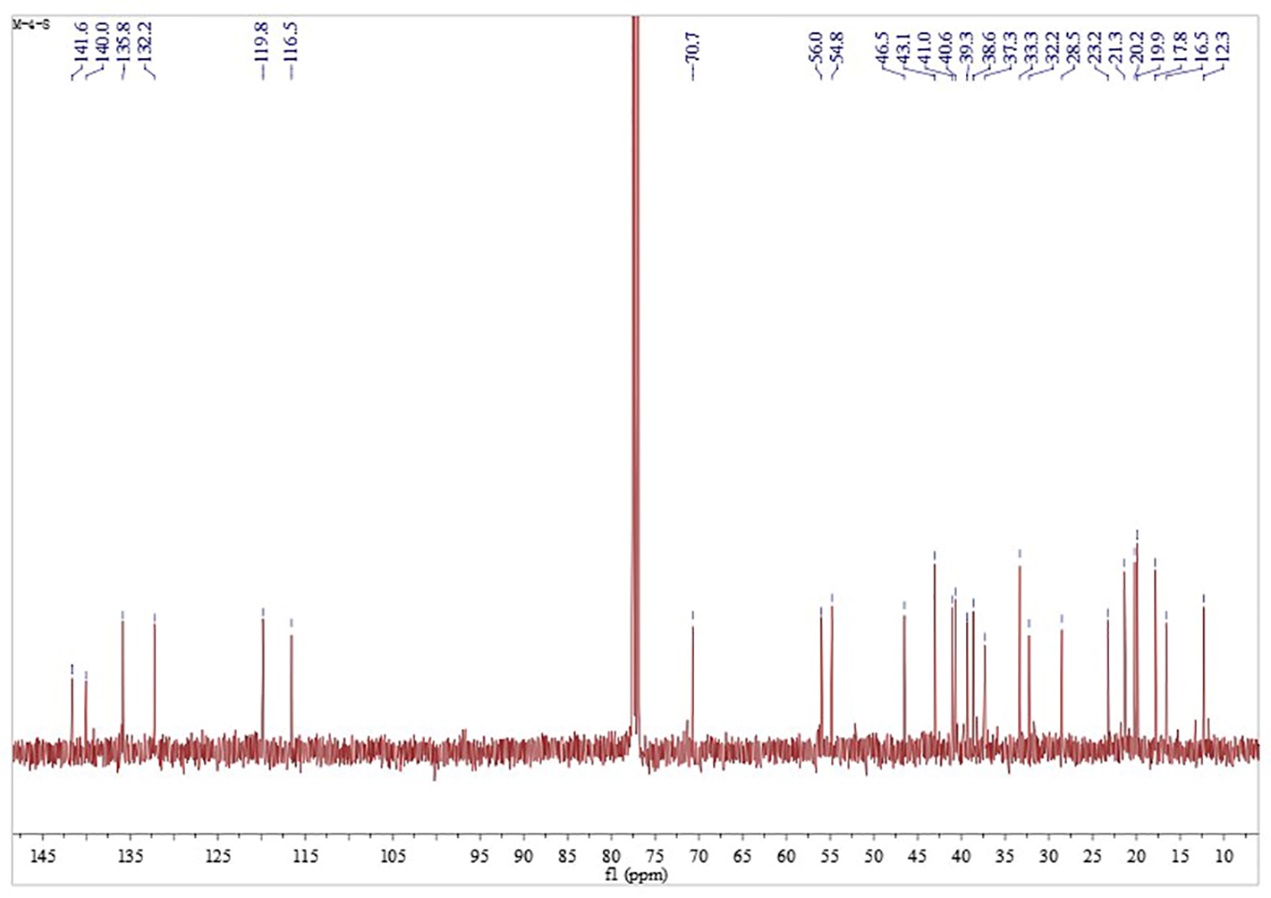


**Figure 17.** ^13^C-NMR spectra of compound **5**.


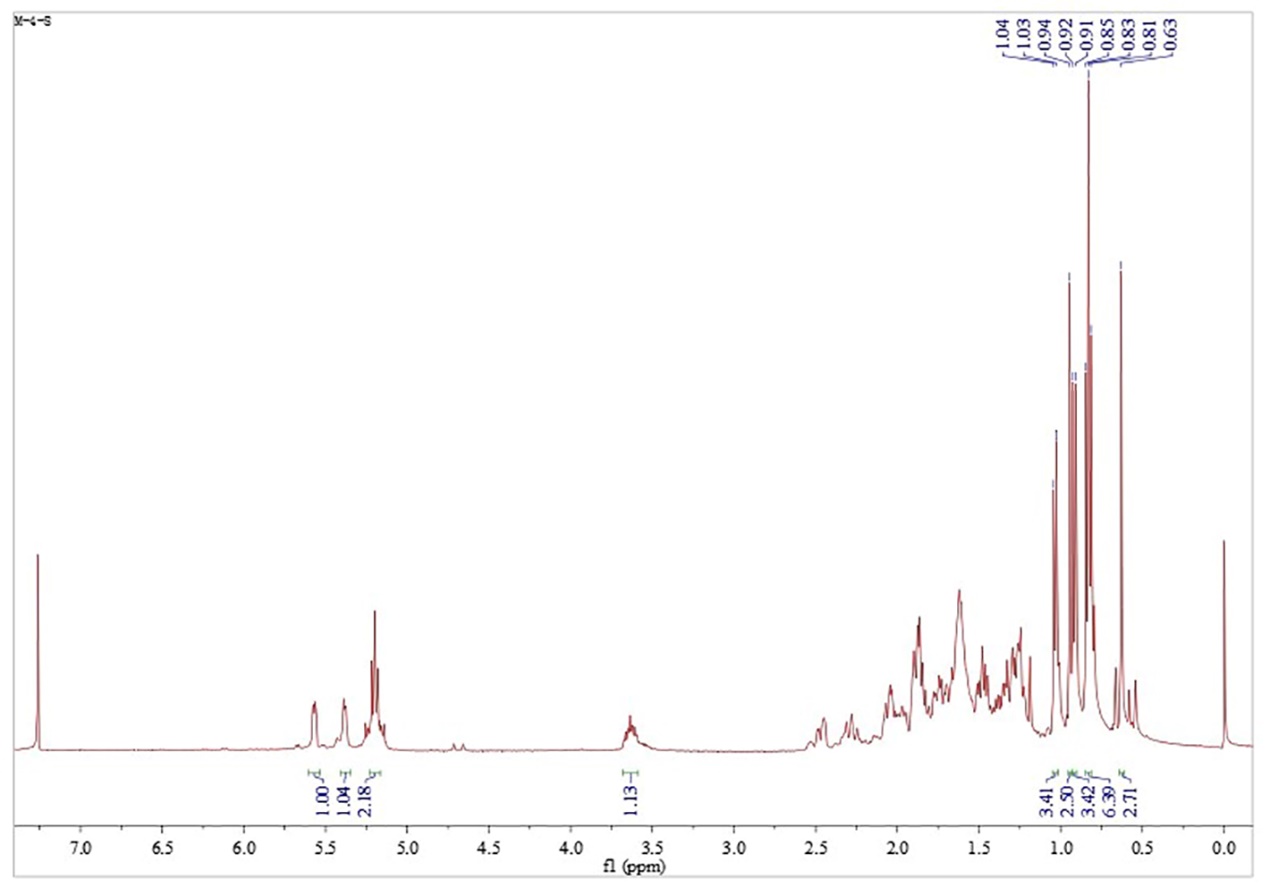


**Figure 18.** ^1^H-NMR spectra of compound **6**.


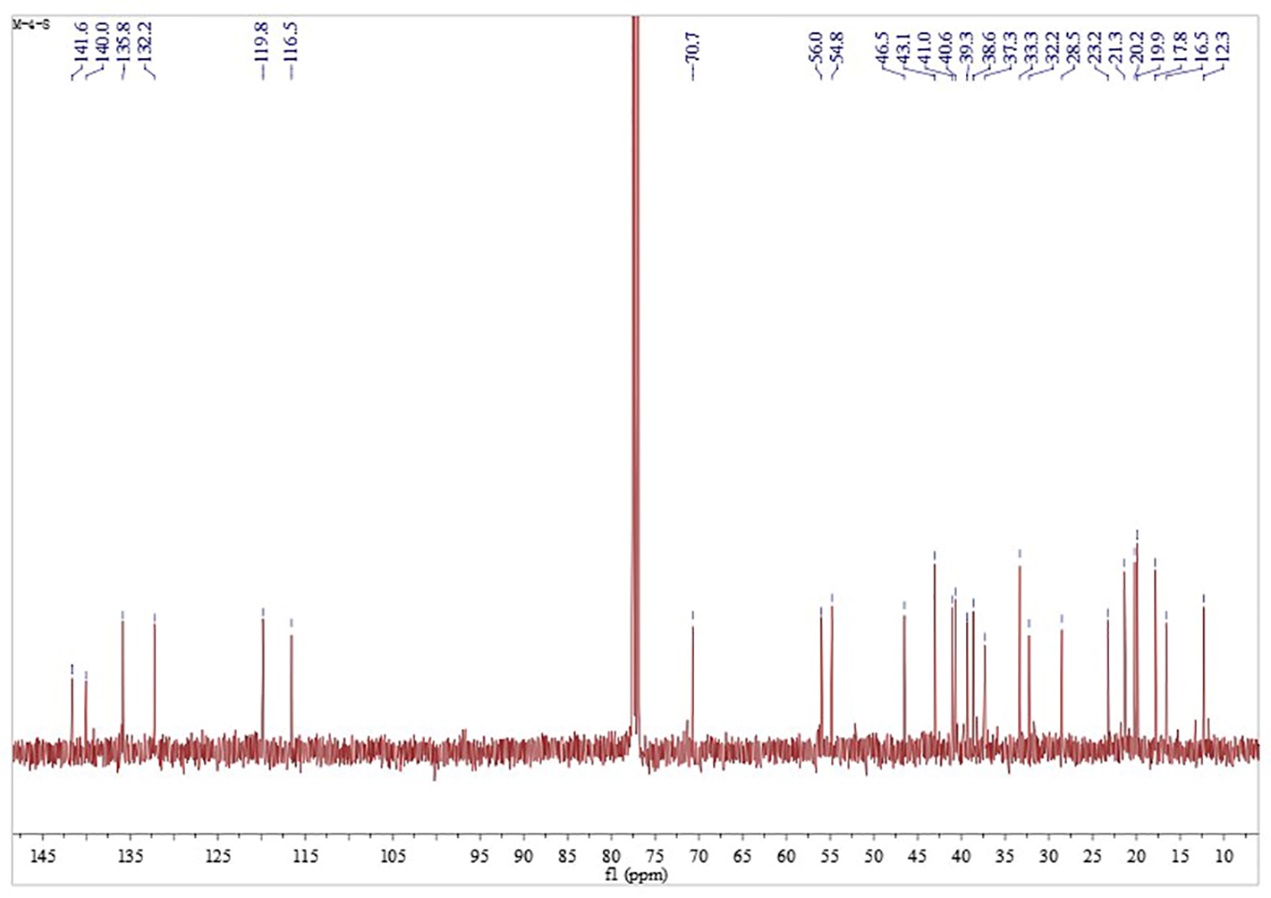


**Figure 19.** ^13^C-NMR spectra of compound **6**.
