# Supplementary figures and images for: Rapid Screening and Identification of Antitumor Ingredients from the Mangrove Endophytic Fungus Using an Enzyme-Immobilized Magnetic Nanoparticulate System
Source: Molecules. 2021 Apr 13;26(8):2255. doi: 10.3390/molecules26082255 (PMC8069786; doi:10.3390/molecules26082255)

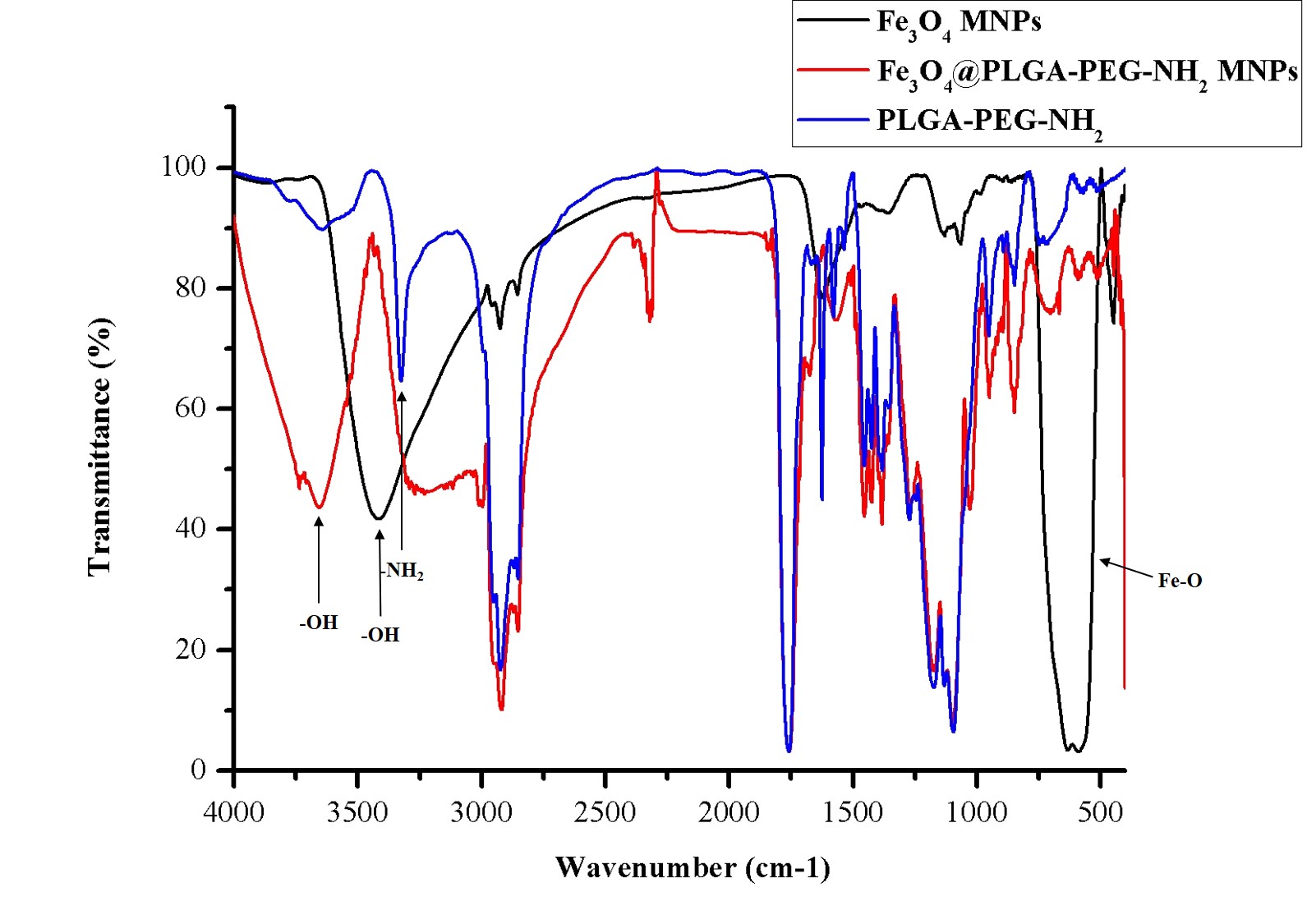

Supplement: Supplementary file 1 [file molecules-26-02255-s001.zip › Supplementary Files/Figure S1.jpg]

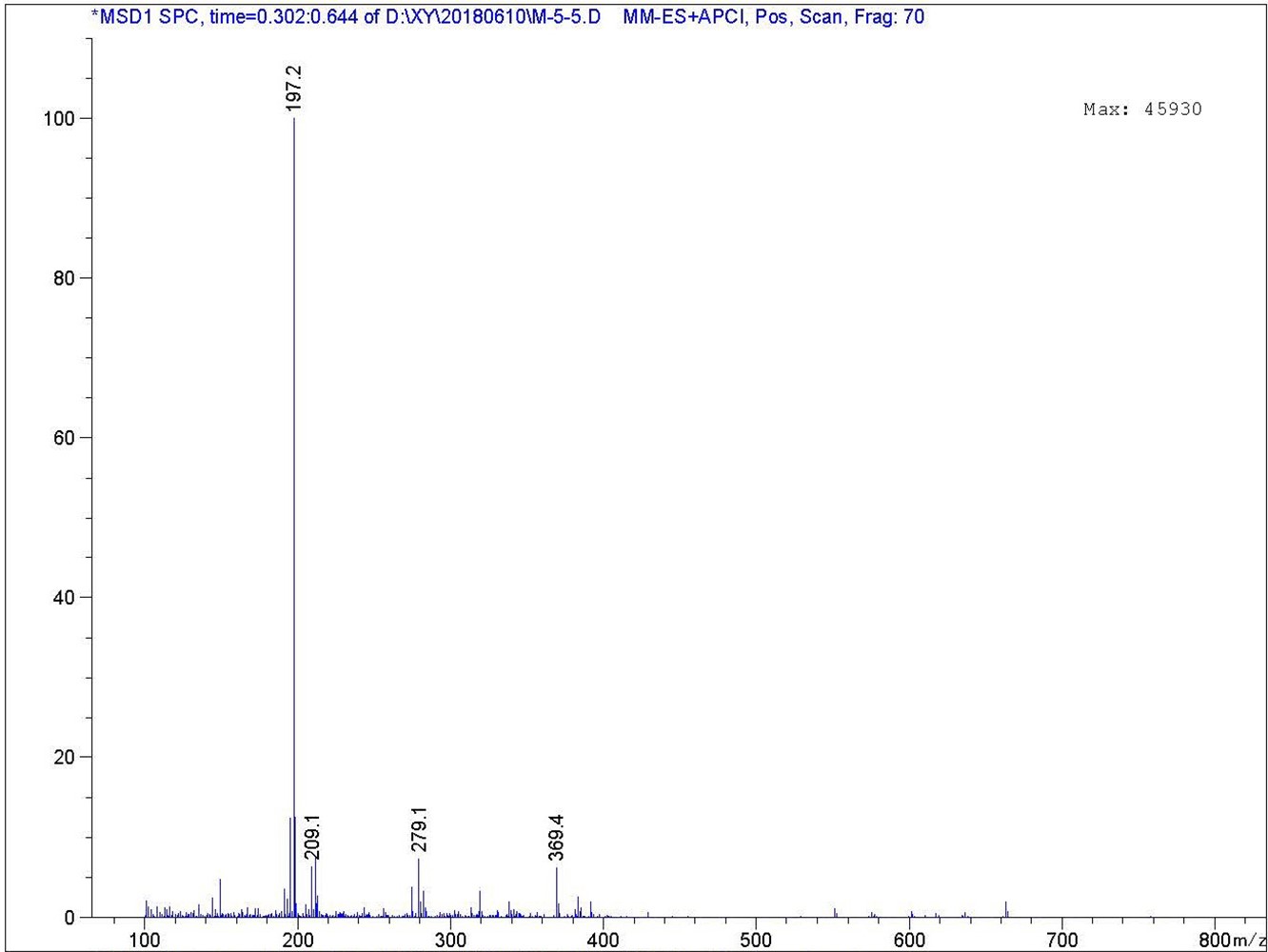

Supplement: Supplementary file 1 [file molecules-26-02255-s001.zip › Supplementary Files/Figure S10.jpg]

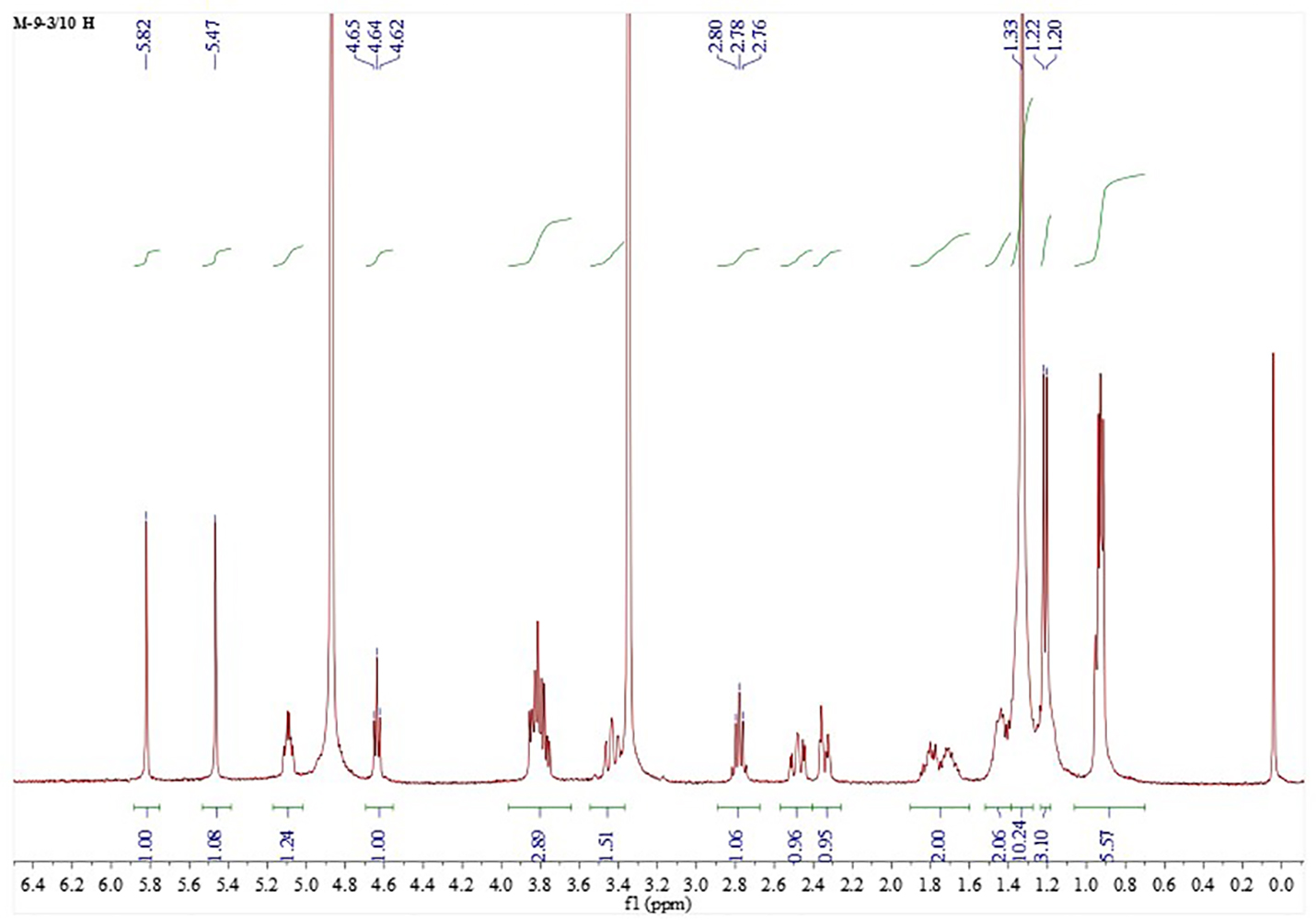

Supplement: Supplementary file 1 [file molecules-26-02255-s001.zip › Supplementary Files/Figure S11.jpg]

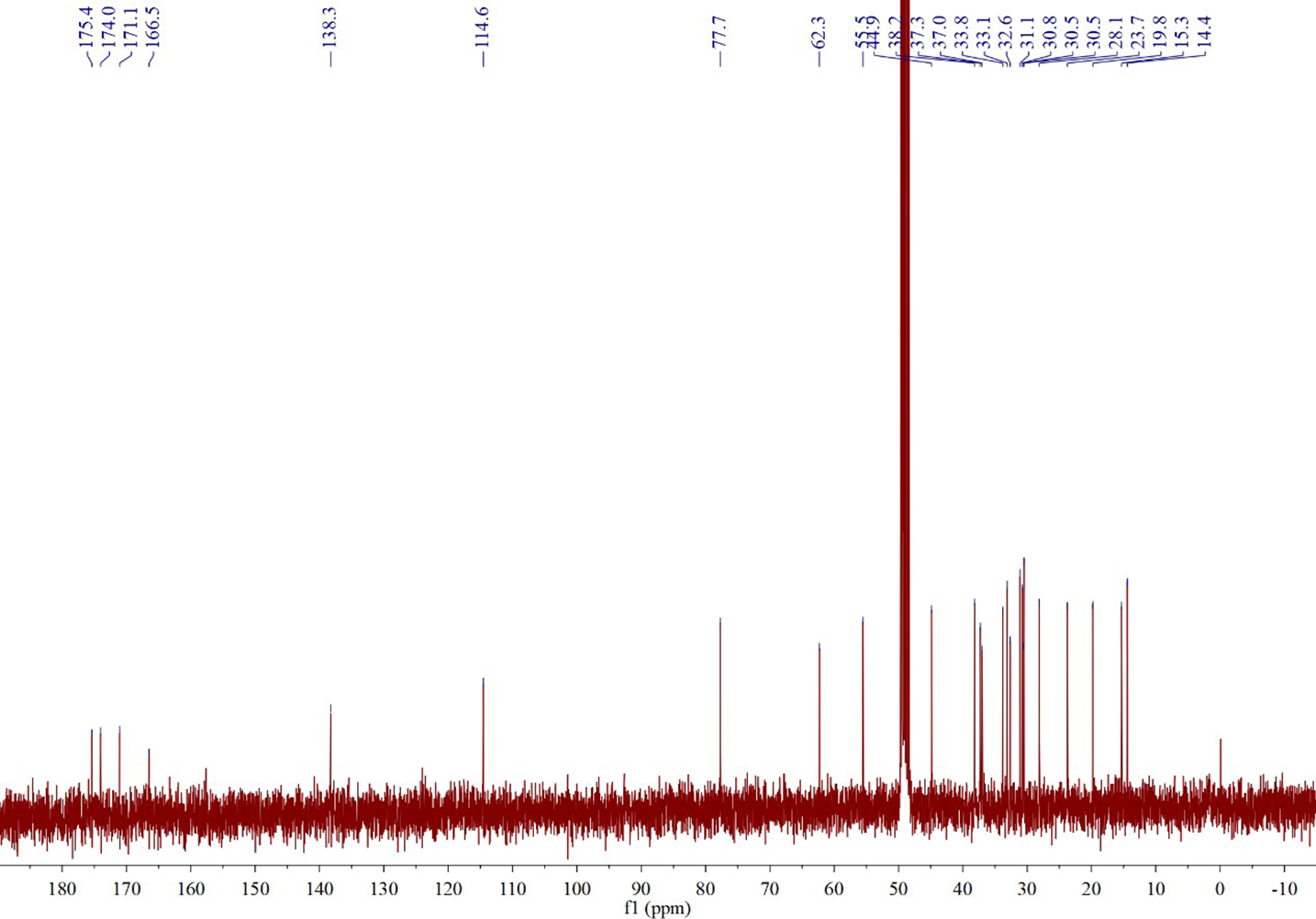

Supplement: Supplementary file 1 [file molecules-26-02255-s001.zip › Supplementary Files/Figure S12.jpg]

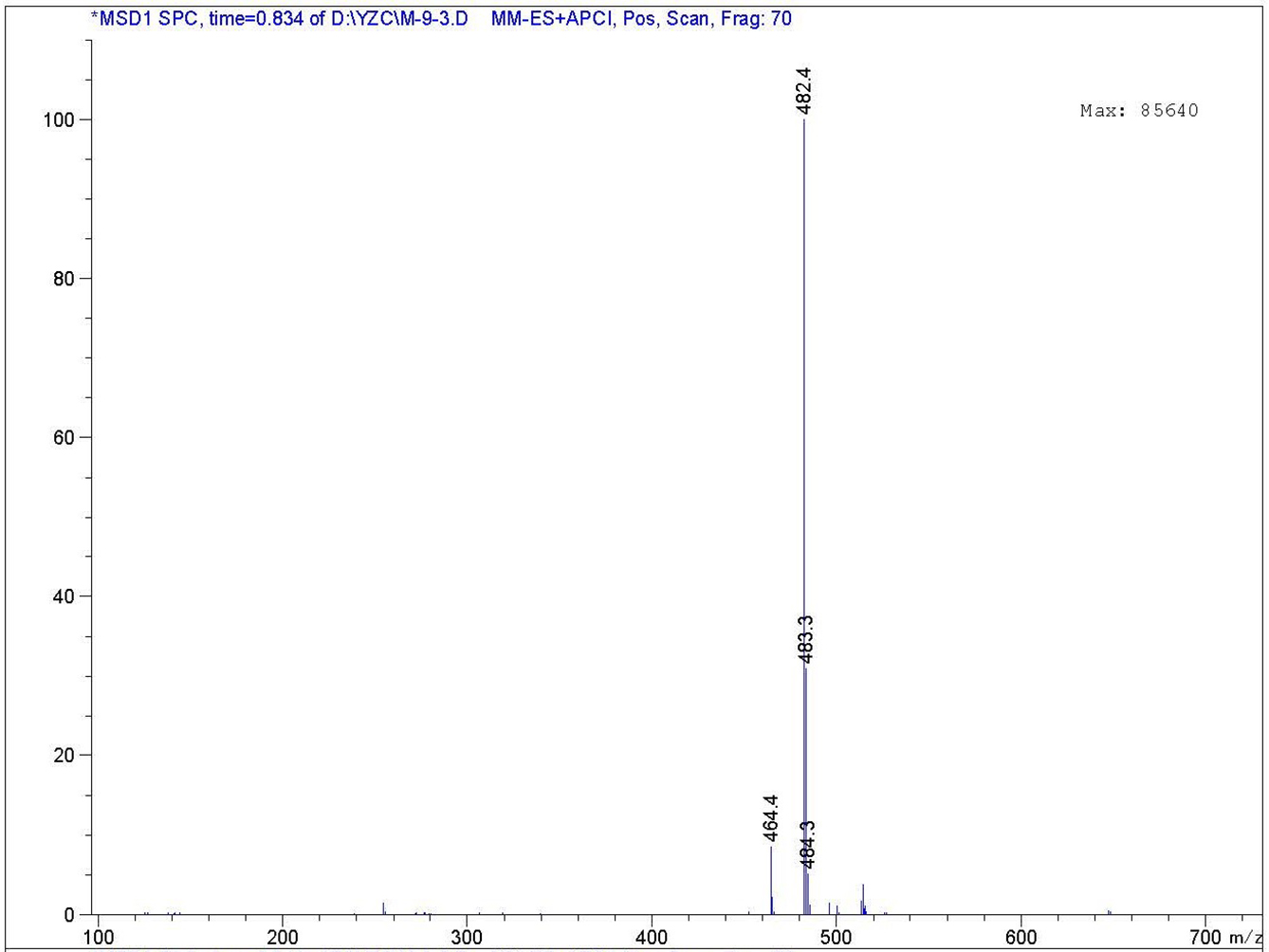

Supplement: Supplementary file 1 [file molecules-26-02255-s001.zip › Supplementary Files/Figure S13.jpg]

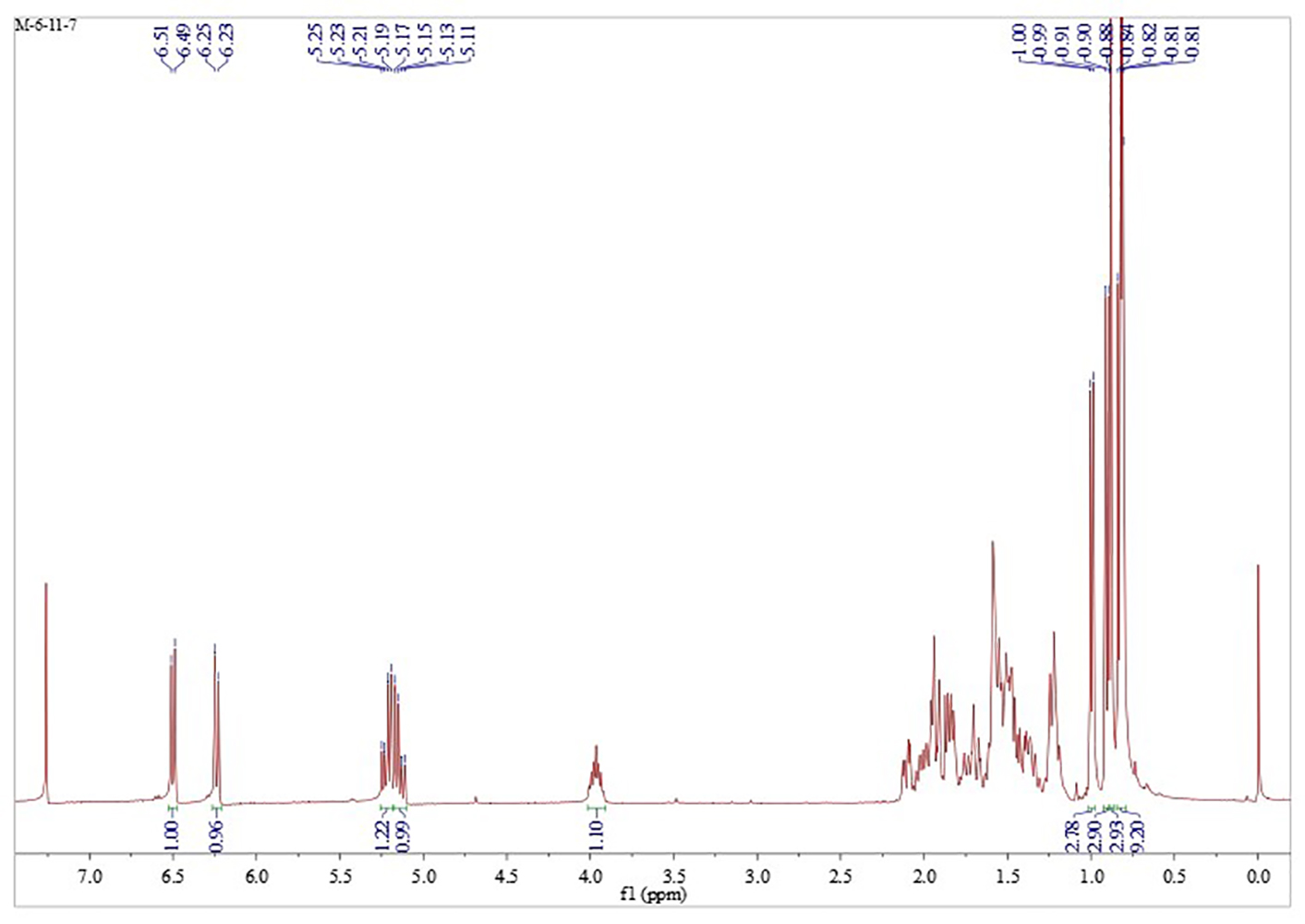

Supplement: Supplementary file 1 [file molecules-26-02255-s001.zip › Supplementary Files/Figure S14.jpg]

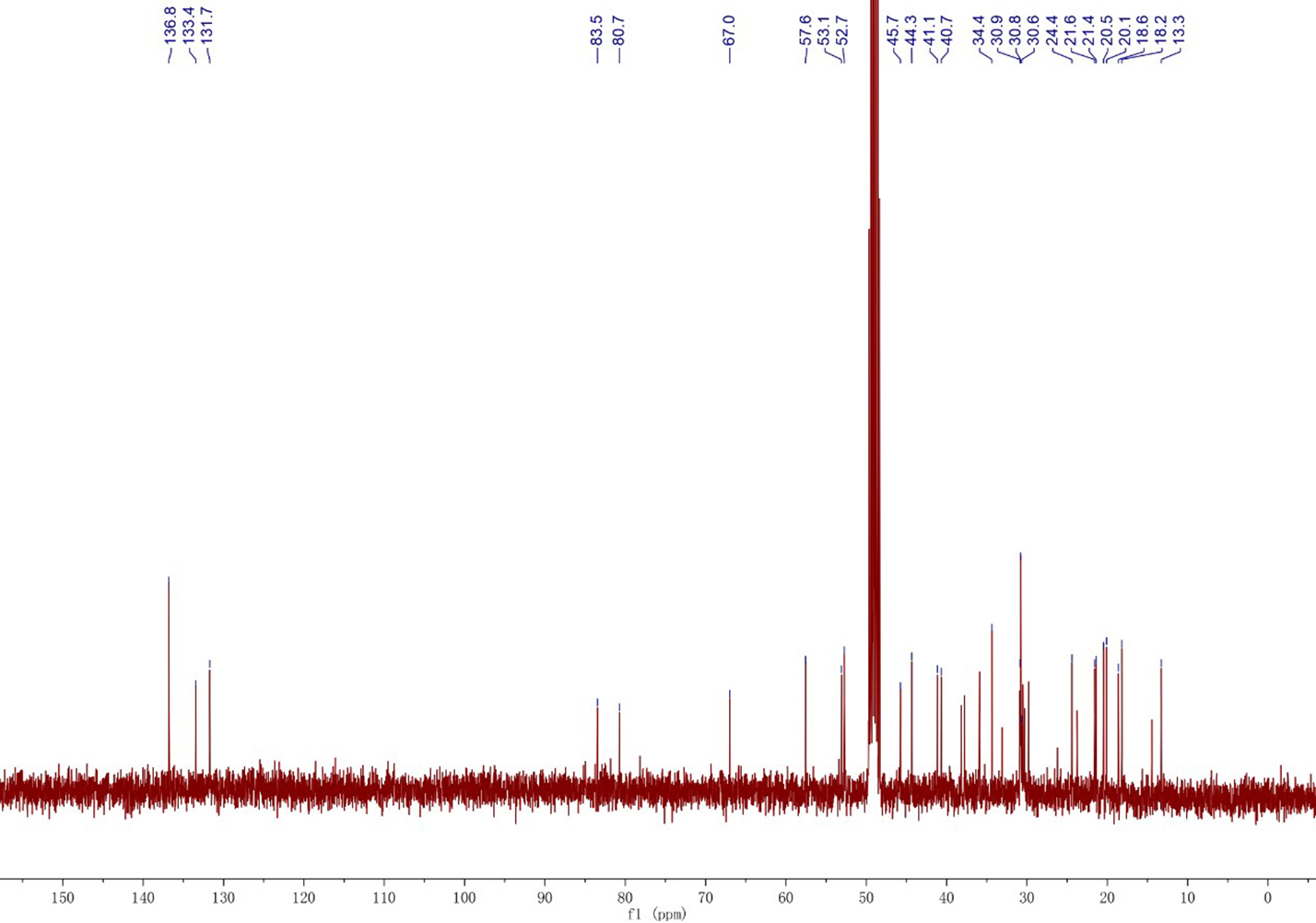

Supplement: Supplementary file 1 [file molecules-26-02255-s001.zip › Supplementary Files/Figure S15.jpg]

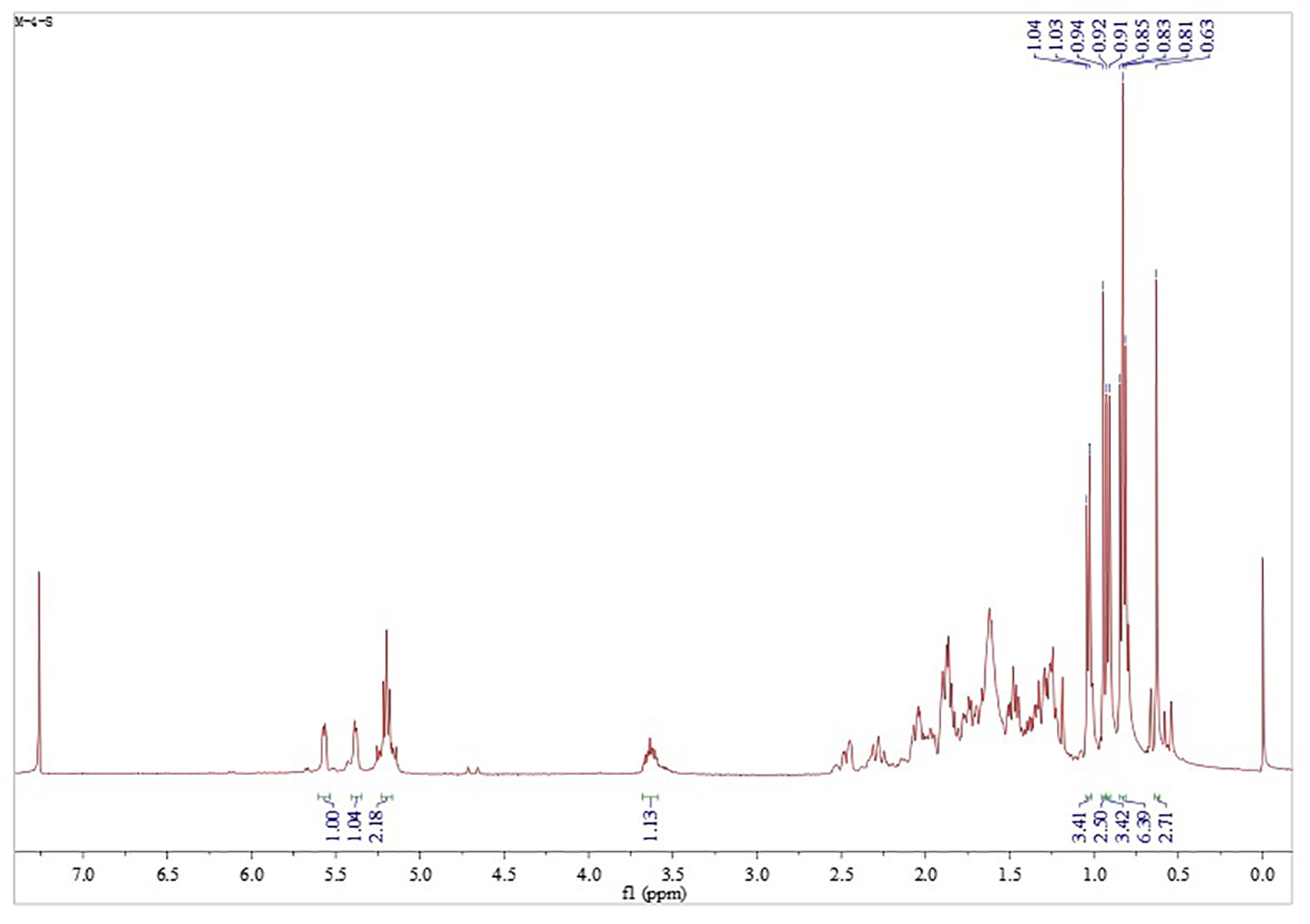

Supplement: Supplementary file 1 [file molecules-26-02255-s001.zip › Supplementary Files/Figure S16.jpg]

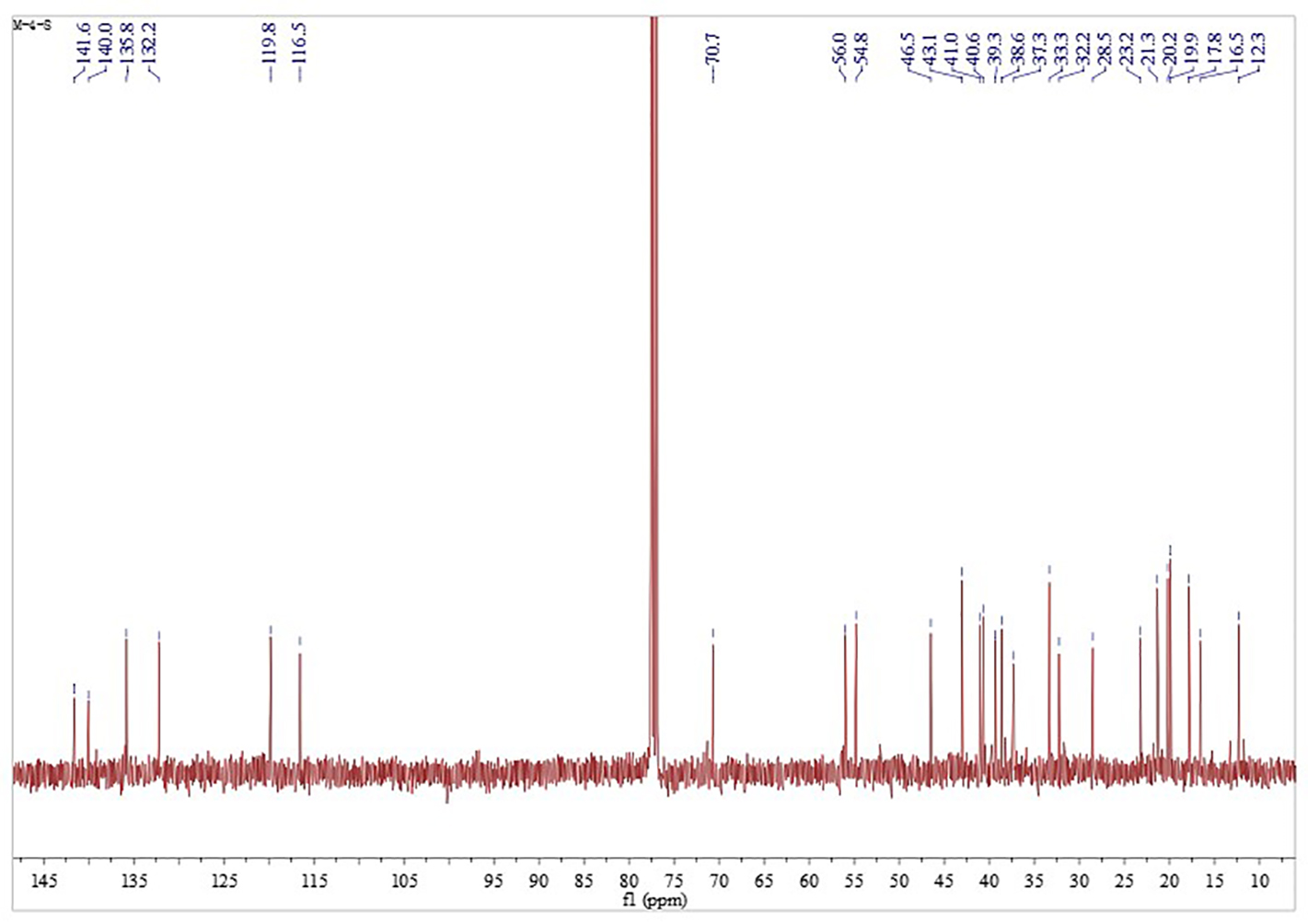

Supplement: Supplementary file 1 [file molecules-26-02255-s001.zip › Supplementary Files/Figure S17.jpg]

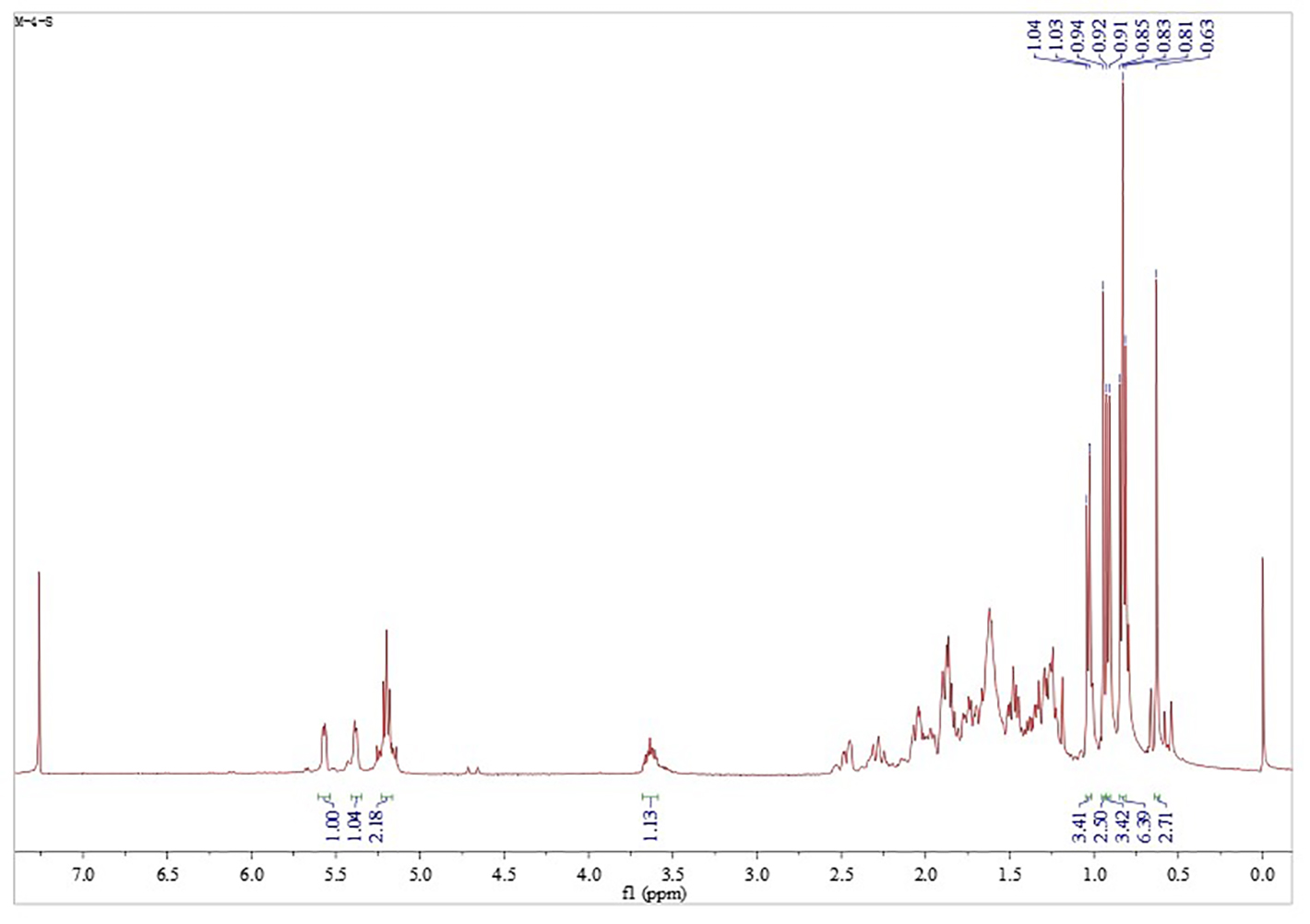

Supplement: Supplementary file 1 [file molecules-26-02255-s001.zip › Supplementary Files/Figure S18.jpg]

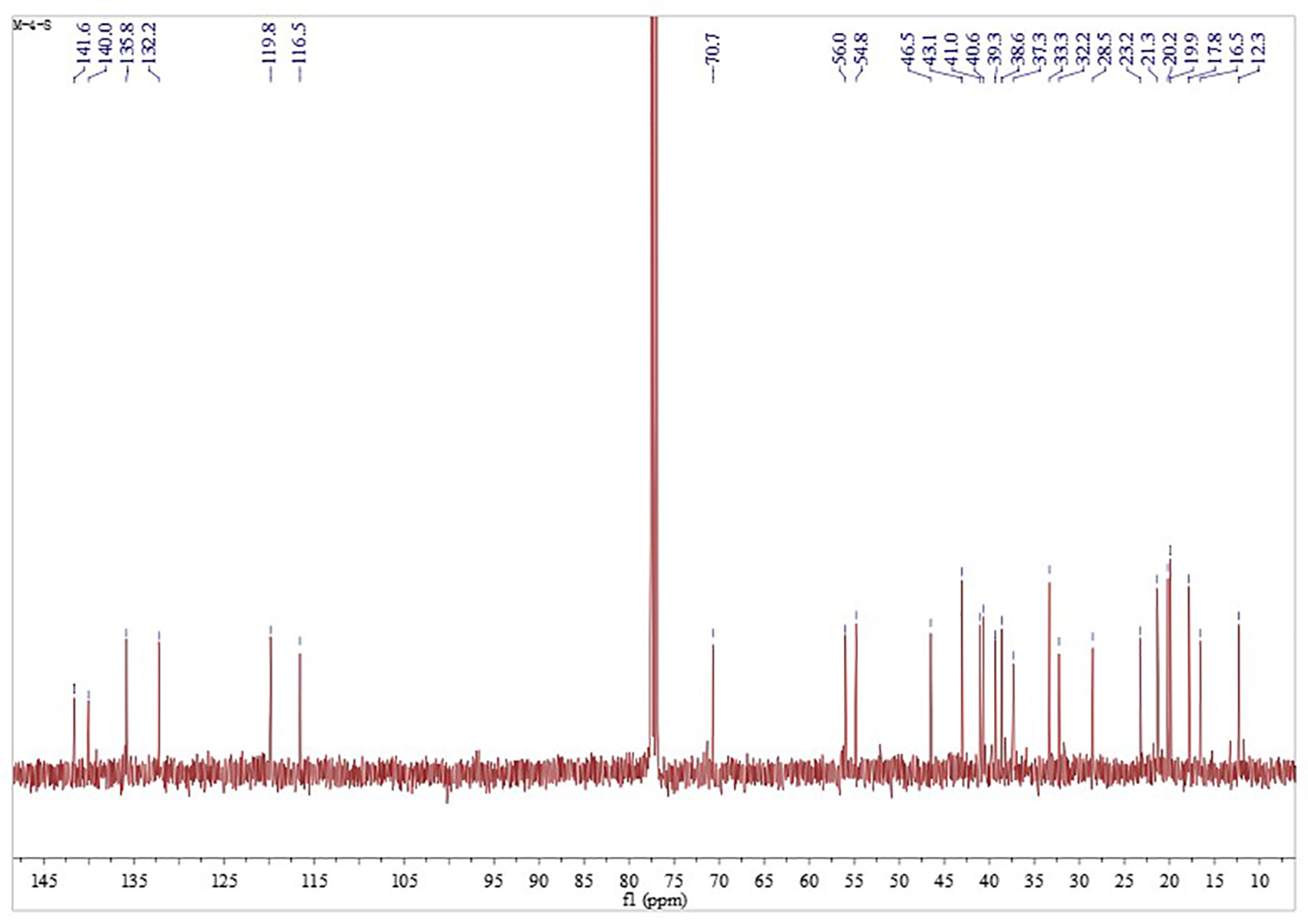

Supplement: Supplementary file 1 [file molecules-26-02255-s001.zip › Supplementary Files/Figure S19.jpg]

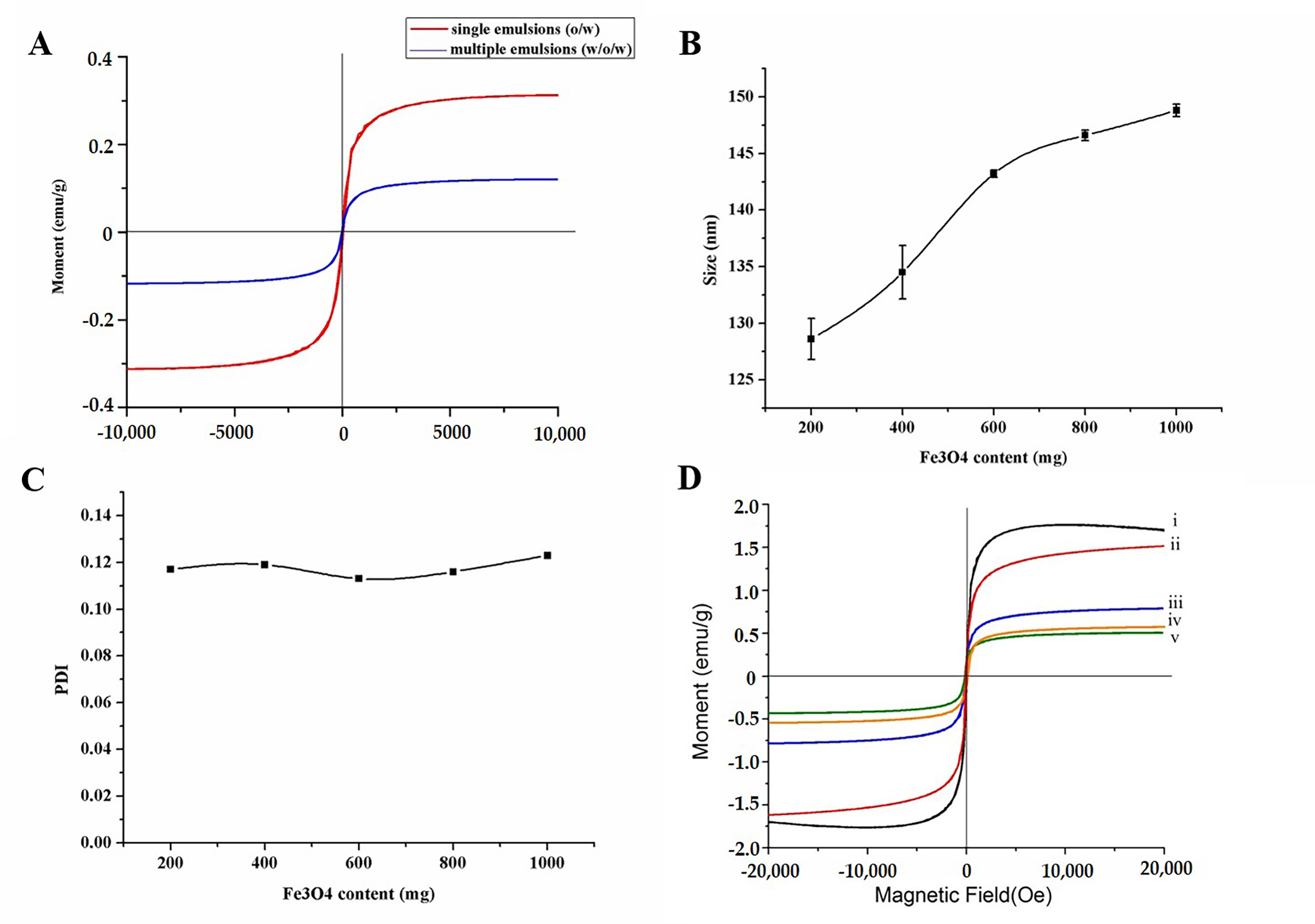

Supplement: Supplementary file 1 [file molecules-26-02255-s001.zip › Supplementary Files/Figure S2.jpg]

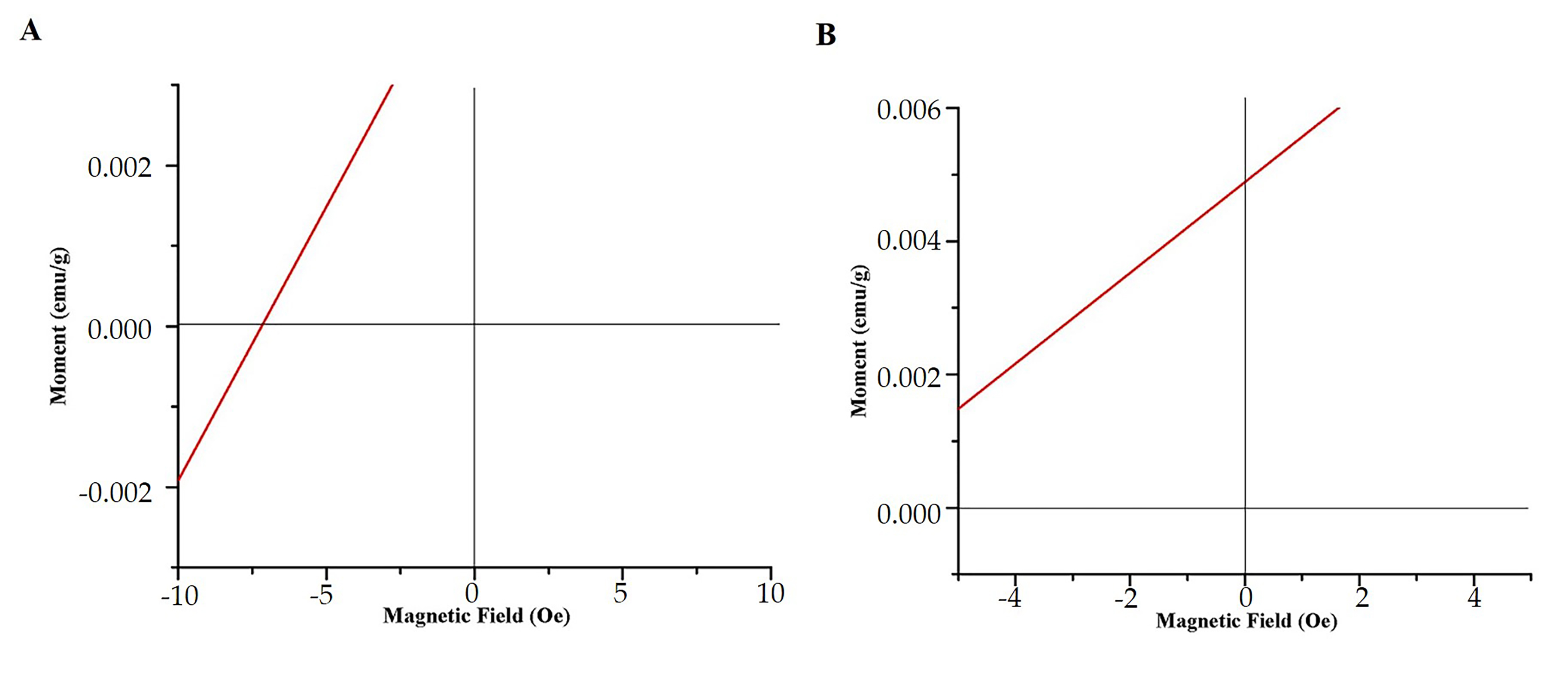

Supplement: Supplementary file 1 [file molecules-26-02255-s001.zip › Supplementary Files/Figure S3.jpg]

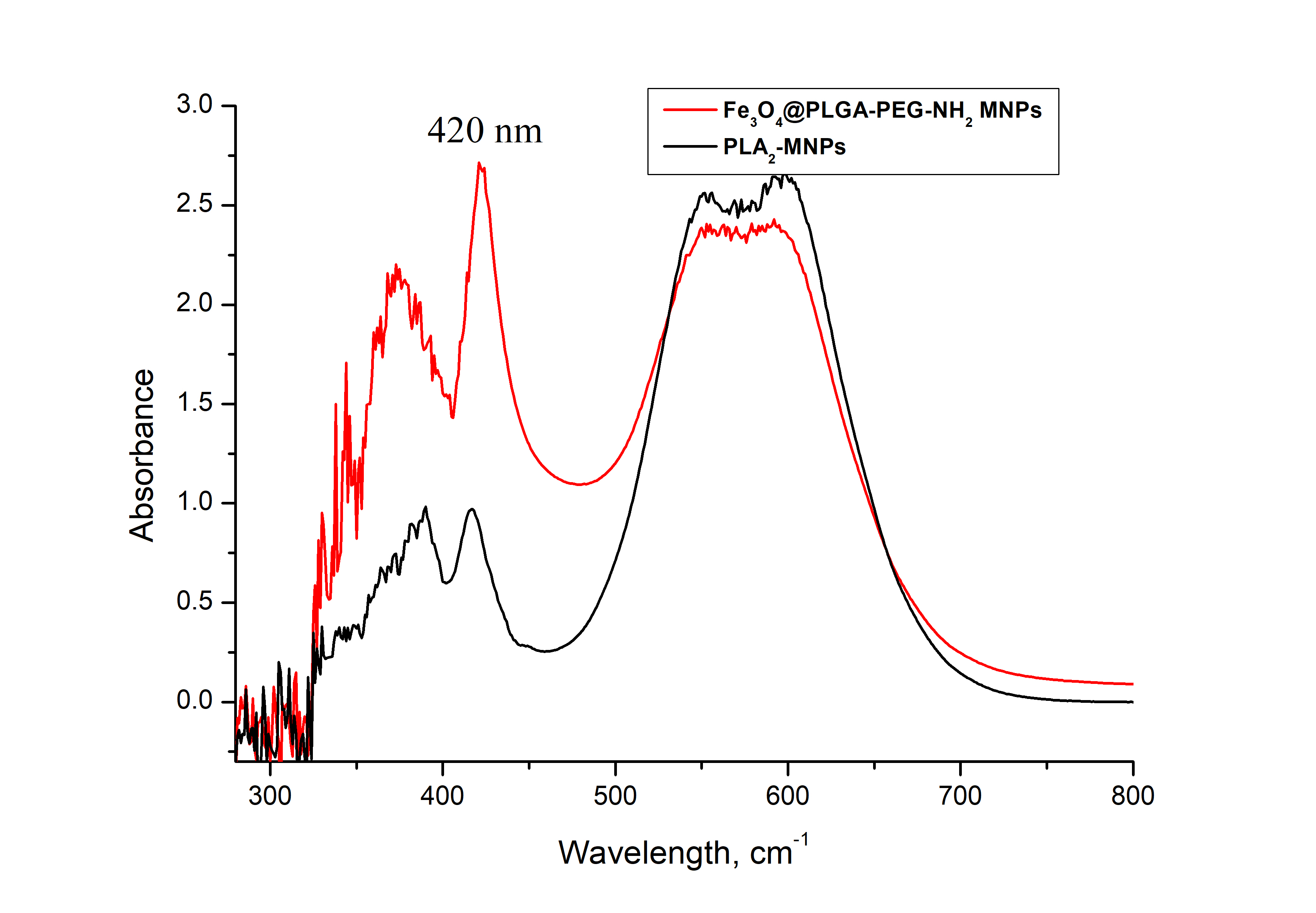

Supplement: Supplementary file 1 [file molecules-26-02255-s001.zip › Supplementary Files/Figure S4.jpg]

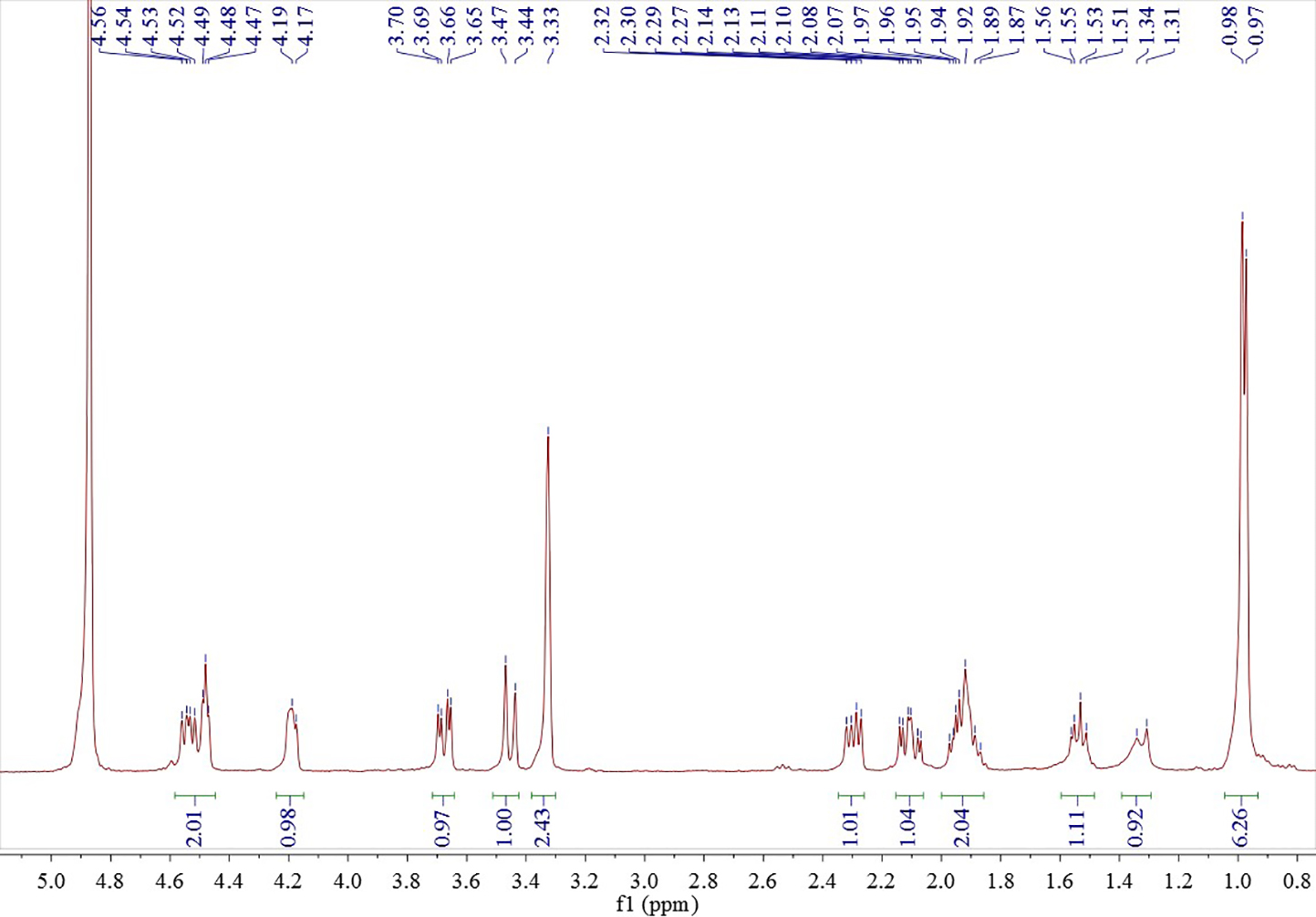

Supplement: Supplementary file 1 [file molecules-26-02255-s001.zip › Supplementary Files/Figure S5.jpg]

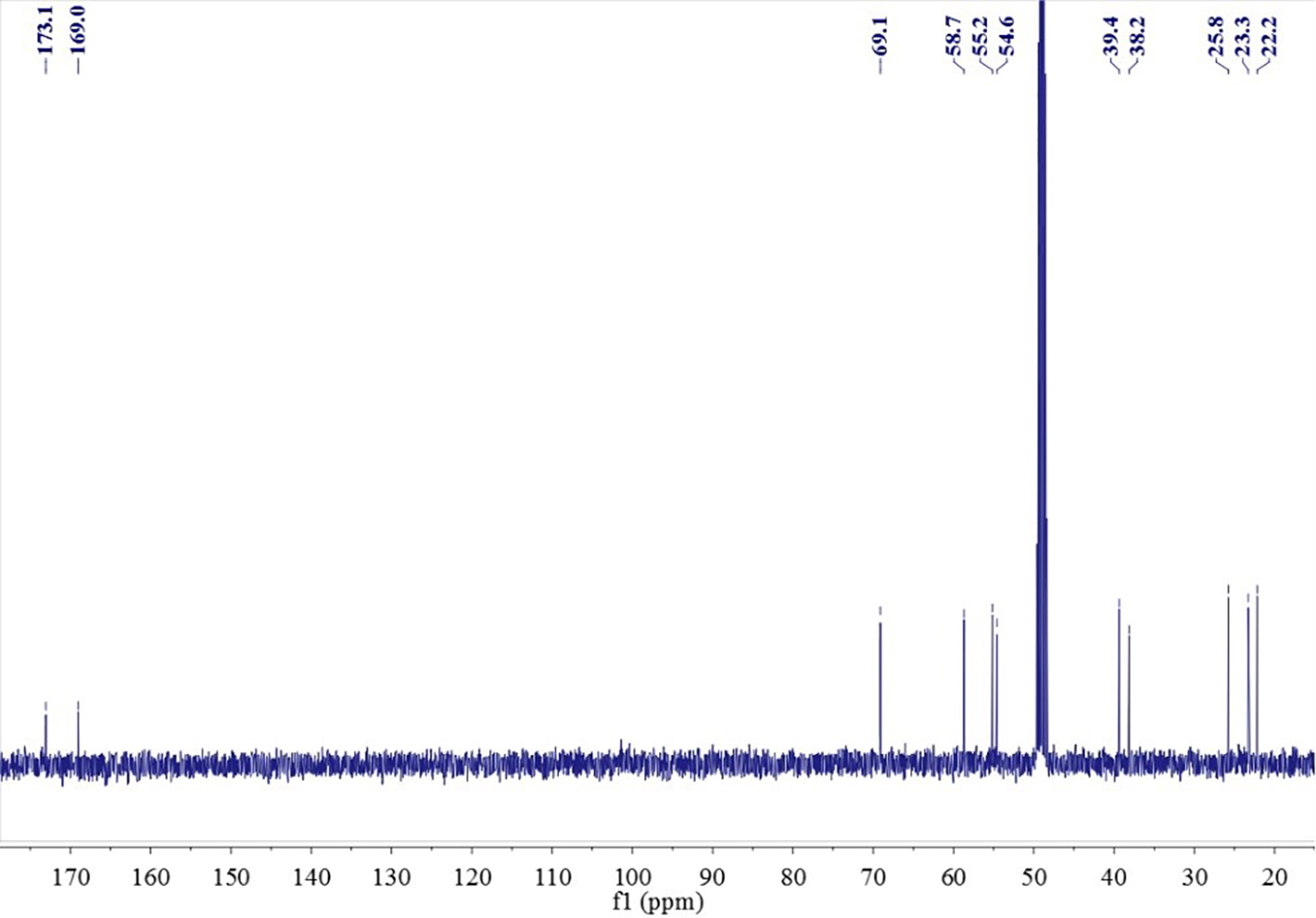

Supplement: Supplementary file 1 [file molecules-26-02255-s001.zip › Supplementary Files/Figure S6.jpg]

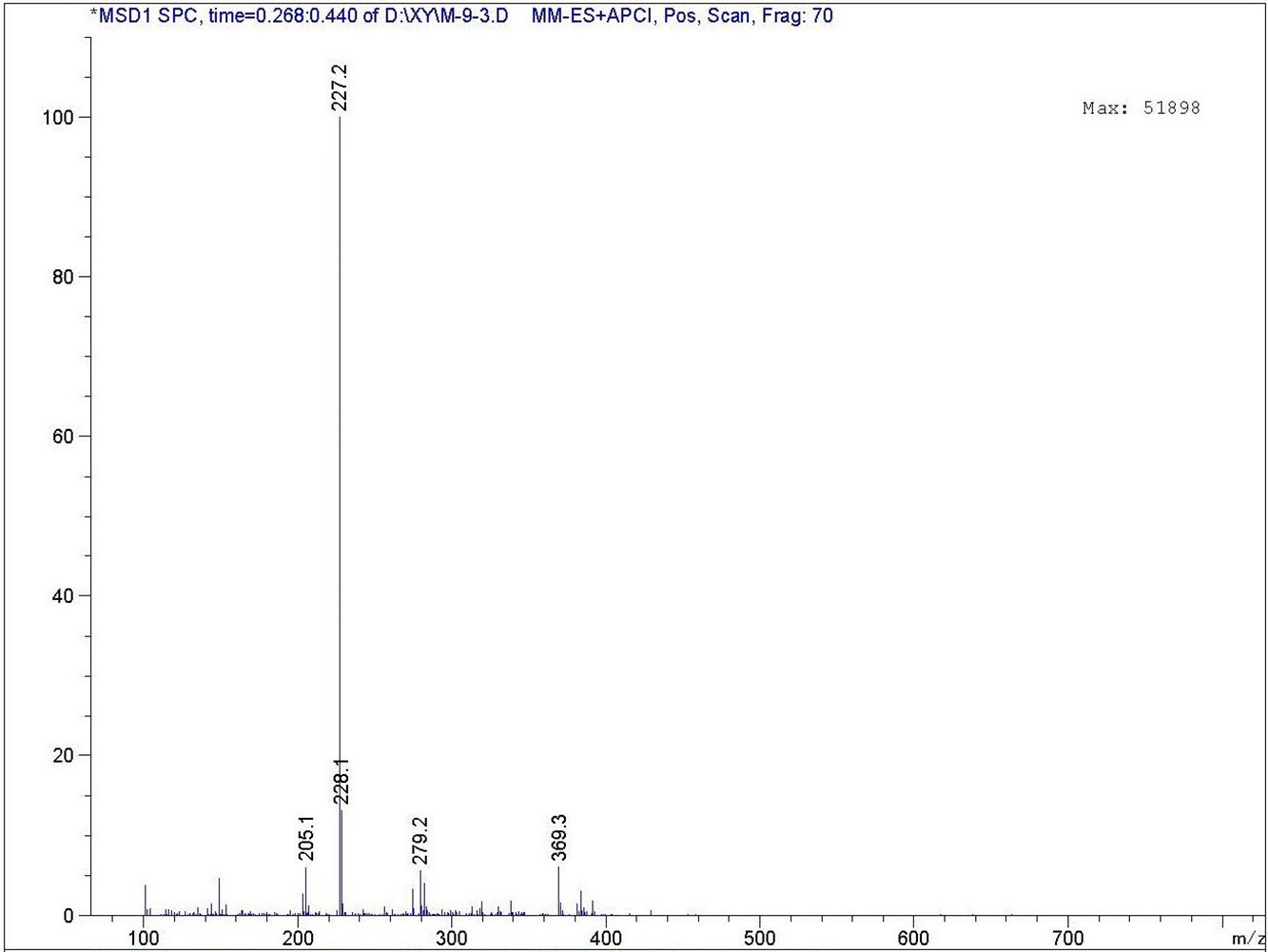

Supplement: Supplementary file 1 [file molecules-26-02255-s001.zip › Supplementary Files/Figure S7.jpg]

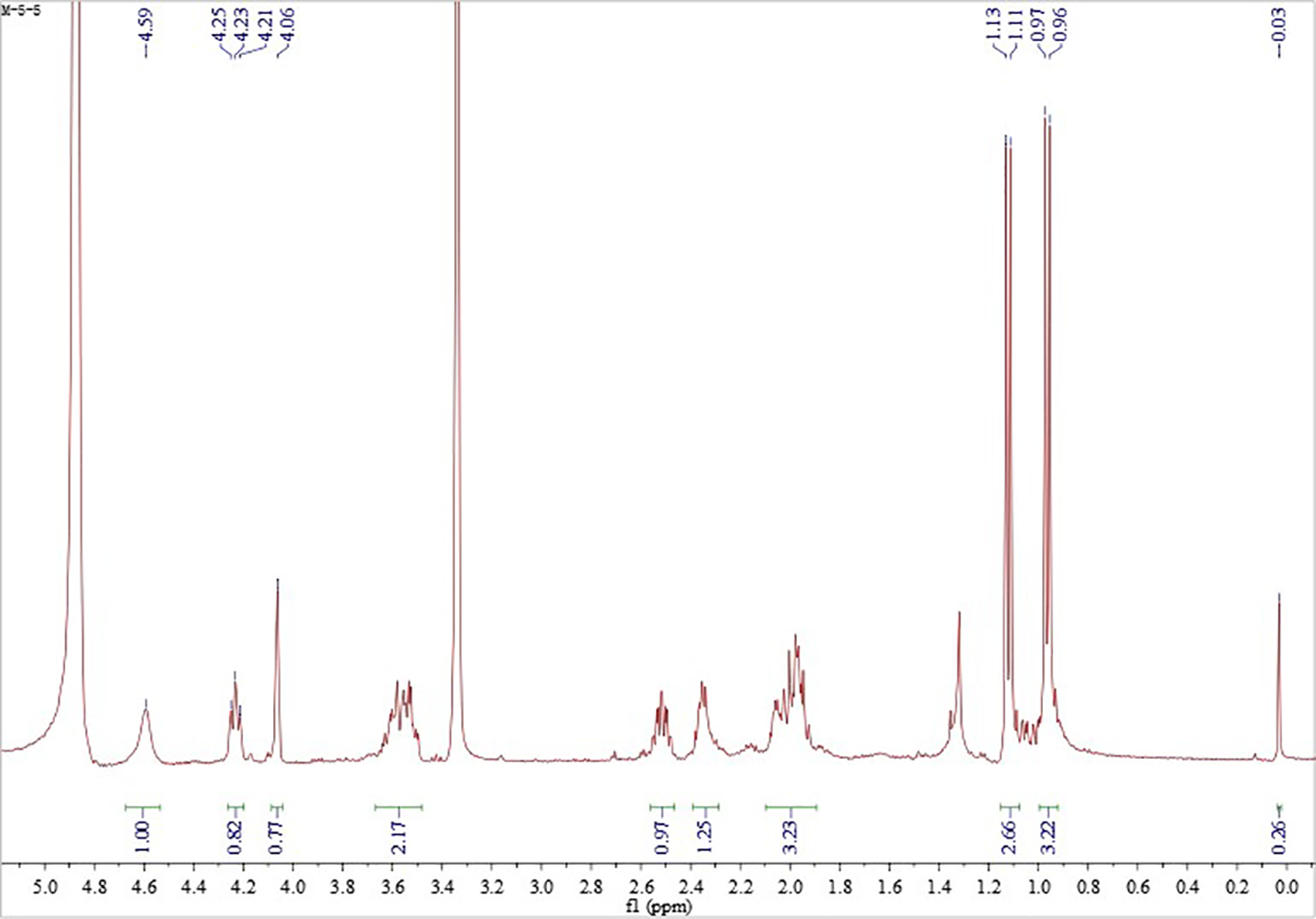

Supplement: Supplementary file 1 [file molecules-26-02255-s001.zip › Supplementary Files/Figure S8.jpg]

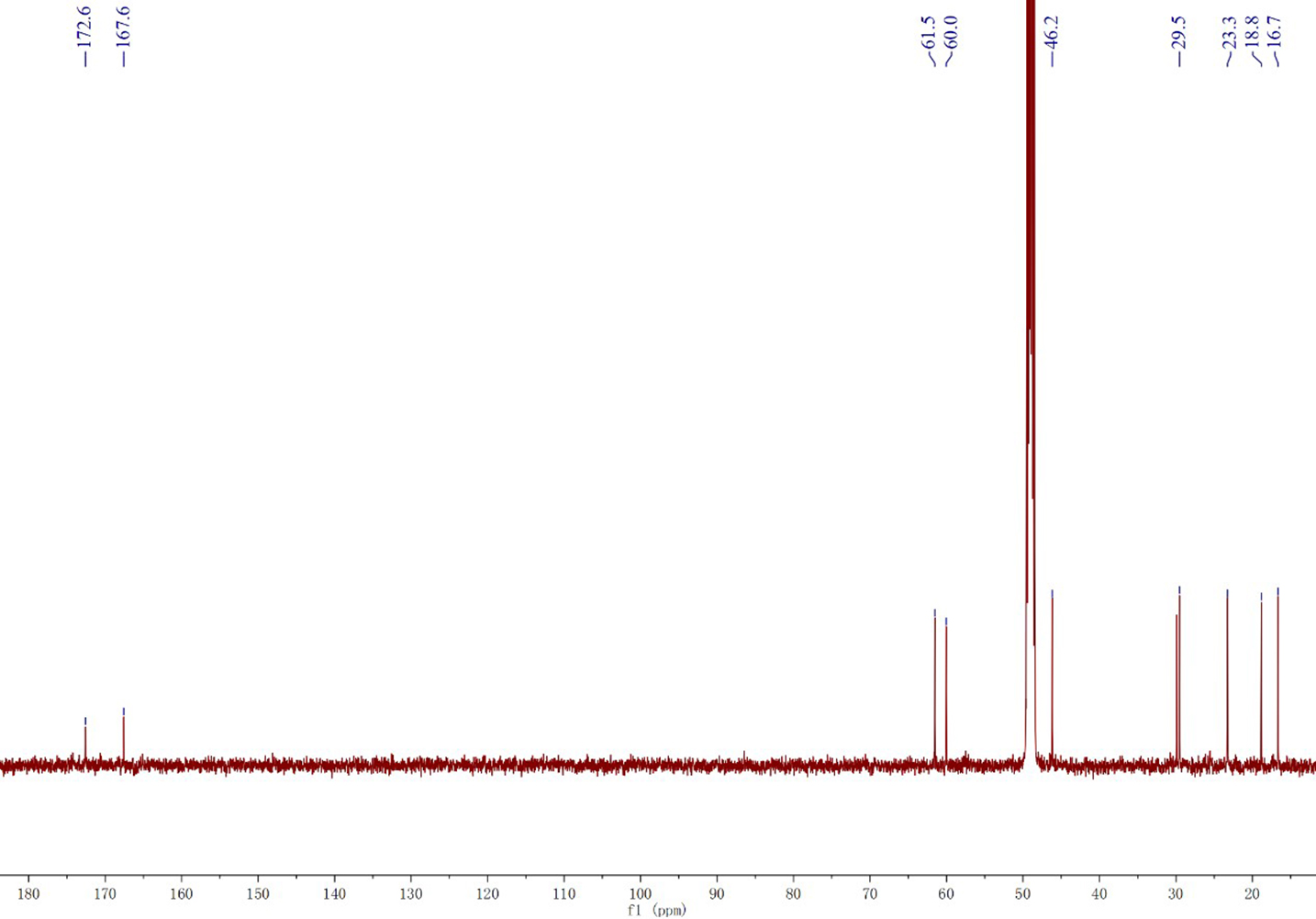

Supplement: Supplementary file 1 [file molecules-26-02255-s001.zip › Supplementary Files/Figure S9.jpg]
